# Supplementary material for: Barriers and facilitators for adopting a healthy lifestyle in a multi-ethnic population: A qualitative study
Source: PLoS One. 2022 Nov 2;17(11):e0277106. doi: 10.1371/journal.pone.0277106 (PMC9629631; doi:10.1371/journal.pone.0277106)
Supplement: S1 File — (DOCX) [file pone.0277106.s001.docx]

**Interview guide: Healthy Lifestyle**

**Defining a Healthy lifestyle**

1. In general, what do you think the phrase ‘healthy lifestyle’ means?
2. Personally, what does a healthy lifestyle mean to you?

**Barriers to Adopting a Healthy Lifestyle**

***Personal barriers***

1. What are some of the personal factors you think people likely face as barriers or challenges to adopting a healthy lifestyle?
2. Why do you think this is a barrier or challenge?
   1. Do you personally face these barriers/challenges as well?
   2. How do you overcome these barriers?
3. Can you think of some things that will help/ make it easier for people to adopt a healthy lifestyle?

***Environmental barriers***

1. What do you feel are some of the Environment factors that act as barriers to adopting a healthy lifestyle?
2. How do you think we can overcome these Environment barriers?

***Cultural & Social barriers***

1. What do you feel are some cultural or social norms and practices in Singapore that act as barriers to having a healthy lifestyle?
   1. In your own community, are there any cultural or social norms and practices that you feel are barriers? Can you tell me more about them? Why do you think they are barriers (if needed)?
2. Does family have an influence on your lifestyle choices and behaviours? Can you tell me more about it?
3. How do you think we can overcome these cultural or social barriers?
4. Do your colleagues at your workplace have a healthy lifestyle? What makes you think that way?
   1. Do you feel your workplace/work affects your lifestyle choices and health? Why or why not?

***Other Barriers***

1. Besides those we have already discussed, are there any other barriers you can think of?
   1. How do you think we can overcome these?

**Enablers of healthy living**

***Personal enablers***

1. What are some of the personal factors you think enable one to lead a healthy lifestyle?
2. Why do you think this is an enabler?
3. Could you share your own personal experiences of how it enabled you to lead a healthy lifestyle?

***Environment enablers***

1. What do you feel are some of the Environment factors that act as enablers to leading a healthy lifestyle?

***Cultural & Social enablers***

1. What do you feel are some cultural or social norms and practices in Singapore that enable people to lead a healthy lifestyle?
   1. In your own community, are there any cultural or social norms and practices that you feel are enablers? How so?

***Technology and Healthy Lifestyle***

1. What is the role of technology (exercise programs in the internet, Apps, etc.) in promoting healthy lifestyle?
2. Do you feel that technology supports a healthy lifestyle in Singapore? How so?
3. On the other hand, do you think technology prevents us from leading a healthy lifestyle? Can you tell us more about it?

***Government Initiatives and Healthy Lifestyle***

1. What do you think about the ongoing healthy lifestyle initiatives in Singapore that are run by government?
2. What are some of the benefits or drawbacks of such initiatives?
3. Can you think of any other initiatives/programmes that can be implemented in your community to make it easier for people to lead a healthy lifestyle? What would you like to see implemented in the future?
