# Supplementary material for: Barriers and facilitators for adopting a healthy lifestyle in a multi-ethnic population: A qualitative study
Source: PLoS One. 2022 Nov 2;17(11):e0277106. doi: 10.1371/journal.pone.0277106 (PMC9629631; doi:10.1371/journal.pone.0277106)
Supplement: Attachment — Submitted filename: PONE-D-22-13181_reviewer_JRH.pdf [file pone.0277106.s002.pdf]

PLOS ONE

Barriers and Facilitators for adopting a healthy lifestyle in a multi-ethnic population: A qualitative study

--Manuscript Draft--

**Manuscript Number:**	PONE-D-22-13181
**Article Type:**	Research Article
**Full Title:**	Barriers and Facilitators for adopting a healthy lifestyle in a multi-ethnic population: A qualitative study
**Short Title:**	Barriers and Facilitators of a Healthy Lifestyle
**Corresponding Author:**	Mythily Subramaniam<br>Institute of Mental Health<br>Singapore, SINGAPORE
**Keywords:**	healthy lifestyle; willpower; family; technology; qualitative
**Abstract:**	A healthy lifestyle if defined as 'a way of living that lowers the risk of being seriously ill or dying early.' However, despite the awareness that not adopting a healthy lifestyle can result in long-term harm, a significant proportion of the population do not adopt a healthy lifestyle which increases the risk of developing chronic illnesses in later life. The current study leveraged the socio-ecological model to gain a deeper understanding of the barriers and facilitators of healthy lifestyle in Singapore using a qualitative methodology. In all 30 semi-structured interviews were conducted in English and other local languages. Transcripts were analysed using framework analysis. Five main themes pertaining to personal, interpersonal, environmental, socio-cultural, and policy-level factors were classified under the two overarching themes of barriers and facilitators of healthy lifestyles. The results of this study offer important insights into understanding the barriers and facilitators to the adoption of a healthy lifestyle among people in Singapore. Our findings illustrate the complex interplay between individuals, social relationship, environment, and policy that can act as either a barrier or a facilitator to adopting a healthy lifestyle.
**Order of Authors:**	Mythily Subramaniam
	Fiona Devi
	P.V. AshaRani 1
	Yunjue Zhang
	Peizhi1
	Anitha Jeyagurunathan
	Kumarasan Roystonn
	Janhavi Ajit Vaingankar
	Siow Ann Chong
**Additional Information:**	
**Question**	**Response**
**Financial Disclosure**<br><br>Enter a financial disclosure statement that describes the sources of funding for the work included in this submission. Review the submission guidelines for detailed requirements. View published research articles from *PLOS ONE* for specific examples.	SAC received the funding<br>NMRC/HSRG/0085/2018<br>This study is supported by the Singapore Ministry of Health's National Medical Research Council under its Health Services Research Grant<br>https://www.nmrc.gov.sg/who-we-are<br><br>The funders had no role in study design, data collection and analysis, decision to publish, or preparation of the manuscript.

Powered by Editorial Manager® and ProduXion Manager® from Aries Systems Corporation

This statement is required for submission and **will appear in the published article** if the submission is accepted. Please make sure it is accurate.<br><br>**Unfunded studies**<br>Enter: *The author(s) received no specific funding for this work.*<br><br>**Funded studies**<br>Enter a statement with the following details:<br>• Initials of the authors who received each award<br>• Grant numbers awarded to each author<br>• The full name of each funder<br>• URL of each funder website<br>• Did the sponsors or funders play any role in the study design, data collection and analysis, decision to publish, or preparation of the manuscript?<br>• **NO** - Include this sentence at the end of your statement: *The funders had no role in study design, data collection and analysis, decision to publish, or preparation of the manuscript.*<br>• **YES** - Specify the role(s) played.<br><br>* typeset	
**Competing Interests**<br><br>Use the instructions below to enter a competing interest statement for this submission. On behalf of all authors, disclose any competing interests that could be perceived to bias this work—acknowledging all financial support and any other relevant financial or non-financial competing interests.<br><br>This statement is required for submission and **will appear in the published article** if the submission is accepted. Please make sure it is accurate and that any funding sources listed in your Funding Information later in the submission form are also declared in your Financial Disclosure statement.<br><br>View published research articles from	The authors have declared that no competing interests exist.

Powered by Editorial Manager® and ProduXion Manager® from Aries Systems Corporation

PLOS ONE for specific examples.<br><br>**NO authors have competing interests**<br><br>Enter: *The authors have declared that no competing interests exist.*<br><br>**Authors with competing interests**<br><br>Enter competing interest details beginning with this statement:<br><br>*I have read the journal's policy and the authors of this manuscript have the following competing interests: [insert competing interests here]*<br><br>* typeset	
**Ethics Statement**<br><br>Enter an ethics statement for this submission. This statement is required if the study involved:<br><br>• Human participants<br>• Human specimens or tissue<br>• Vertebrate animals or cephalopods<br>• Vertebrate embryos or tissues<br>• Field research<br><br>Write "N/A" if the submission does not require an ethics statement.<br><br>General guidance is provided below. Consult the submission guidelines for detailed instructions. **Make sure that all information entered here is included in the Methods section of the manuscript.**	Human Subject Research<br>National Healthcare Group Institutional Review Board DSRB Ref: 2019/00926<br>All subjects provided written informed consent

**Format for specific study types**<br><br>**Human Subject Research (involving human participants and/or tissue)**<br>• Give the name of the institutional review board or ethics committee that approved the study<br>• Include the approval number and/or a statement indicating approval of this research<br>• Indicate the form of consent obtained (written/oral) or the reason that consent was not obtained (e.g. the data were analyzed anonymously)<br><br>**Animal Research (involving vertebrate animals, embryos or tissues)**<br>• Provide the name of the Institutional Animal Care and Use Committee (IACUC) or other relevant ethics board that reviewed the study protocol, and indicate whether they approved this research or granted a formal waiver of ethical approval<br>• Include an approval number if one was obtained<br>• If the study involved *non-human primates*, add *additional details* about animal welfare and steps taken to ameliorate suffering<br>• If anesthesia, euthanasia, or any kind of animal sacrifice is part of the study, include briefly which substances and/or methods were applied<br><br>**Field Research**<br><br>Include the following details if this study involves the collection of plant, animal, or other materials from a natural setting:<br>• Field permit number<br>• Name of the institution or relevant body that granted permission	
**Data Availability**<br><br>Authors are required to make all data underlying the findings described fully available, without restriction, and from the time of publication. PLOS allows rare exceptions to address legal and ethical concerns. See the PLOS Data Policy and FAQ for detailed information.	No - some restrictions will apply

A Data Availability Statement describing where the data can be found is required at submission. Your answers to this question constitute the Data Availability Statement and **will be published in the article**, if accepted.<br><br>**Important:** Stating 'data available on request from the author' is not sufficient. If your data are only available upon request, select 'No' for the first question and explain your exceptional situation in the text box.<br><br>Do the authors confirm that all data underlying the findings described in their manuscript are fully available without restriction?	
**Describe where the data may be found in full sentences. If you are copying our sample text, replace any instances of XXX with the appropriate details.**<br><br>• If the data are **held or will be held in a public repository**, include URLs, accession numbers or DOIs. If this information will only be available after acceptance, indicate this by ticking the box below. For example: *All XXX files are available from the XXX database (accession number(s) XXX, XXX.*).<br>• If the data are all contained **within the manuscript and/or Supporting Information files**, enter the following: *All relevant data are within the manuscript and its Supporting Information files.*<br>• If neither of these applies but you are able to provide **details of access elsewhere**, with or without limitations, please do so. For example:<br><br>*Data cannot be shared publicly because of [XXX]. Data are available from the XXX Institutional Data Access / Ethics Committee (contact via XXX) for researchers who meet the criteria for access to confidential data.*<br><br>*The data underlying the results presented in the study are available from (include the name of the third party*	Data cannot be shared publicly because of ethical and institutional regulations. Data are available from the Institute of Mental Health Institutional Research Review Committee (contact via research@imh.com.sg) for researchers who meet the criteria for access to confidential data.

Powered by Editorial Manager® and ProduXion Manager® from Aries Systems Corporation

and contact information or URL).<br>• This text is appropriate if the data are owned by a third party and authors do not have permission to share the data.<br><br>* typeset	
Additional data availability information:	Tick here if your circumstances are not covered by the questions above and you need the journal’s help to make your data available.

Powered by Editorial Manager® and ProduXion Manager® from Aries Systems Corporation

Manuscript

Click here to access/download;Manuscript;Barriers and facilitators PLOSOne.docx

Title: Barriers and Facilitators for adopting a healthy lifestyle in a multi-ethnic population: A qualitative study

Authors: Mythily Subramaniam[1,2], Fiona Devi[1], P.V. AshaRani [1], Yunjue Zhang[1], Peizhi[1], Anitha Jeyagurunathan[1], Kumarasan Roystonn[1], Janhavi Ajit Vaingankar[1], Siow Ann Chong[1]

[1]Research Division, Institute of Mental Health, Singapore

[2]Saw Swee Hock School of Public Health, National University of Singapore

Corresponding Author: Mythily Subramaniam

Assistant Chairman Medical Board (Research)

Research Division, Institute of Mental Health, Buangkok Green Medical Park, 10 Buangkok View, Singapore 539747,

Tel: 65-63893633 Fax: 65-6343 7962, Mythily@imh.com.sg

Abstract

A healthy lifestyle if defined as ’a way of living that lowers the risk of being seriously ill or dying early.’ However, despite the awareness that not adopting a healthy lifestyle can result in long-term harm, a significant proportion of the population do not adopt a healthy lifestyle which increases the risk of developing chronic illnesses in later life. The current study leveraged the socio-ecological model to gain a deeper understanding of the barriers and facilitators of healthy lifestyle in Singapore using a qualitative methodology. In all 30 semi-structured interviews were conducted in English and other local languages. Transcripts were analysed using framework analysis. Five main themes pertaining to personal, interpersonal, environmental, socio-cultural, and policy-level factors were classified under the two overarching themes of barriers and facilitators of healthy lifestyles. The results of this study offer important insights into understanding the barriers and facilitators to the adoption of a healthy lifestyle among people in Singapore. Our findings illustrate the complex interplay between individuals, social relationship, environment, and policy that can act as either a barrier or a facilitator to adopting a healthy lifestyle.

Key words: healthy lifestyle; willpower; family; technology; qualitative

1. Introduction

Life expectancy at birth has increased globally, and improvements in population health status have been observed for every life stage. These changes have been driven by public health initiatives such as universal immunisation, improved health infrastructure and maternal and infant health, tobacco control, and motor vehicle safety [1]. This increased life expectancy is associated with changing disease patterns, i.e., a transition from acute to chronic diseases as the primary source of morbidity and mortality worldwide [2] At the same time, the physician-patient relationship has progressively moved towards shared decision-making 'where clinicians and patients make decisions together using the best available evidence' [3]. With patients becoming avid consumers of medical knowledge available in the public domain, the lay population is expected to assume greater responsibility for their health to prevent and manage their chronic disorders. It is also widely known that many chronic conditions like diabetes, coronary artery diseases, and cancers are potentially preventable through the adoption of a healthy lifestyle such as regular exercise, a good diet, and avoidance of smoking and substance use [4,5].

The World Health Organisation (WHO) defines a healthy lifestyle as 'a way of living that lowers the risk of being seriously ill or dying early' [6]. However, despite the awareness that not adopting a healthy lifestyle can result in long-term harm, a significant proportion of the population do things that they perceive to be easy and or "feel good" in the here and now, which leads to the development of chronic illnesses in later life. Pender's health promotion model defines barriers to a healthy lifestyle as 'factors that directly interfere with the enactment of a health-promoting behavior or mediate by reducing the commitment to the plan of action for changing behavior' [7]. On the other hand, factors that promote or enable the uptake of a healthy lifestyle are referred to as facilitators.

A systematic review of 32 studies by Murray et al. [8] found that ‘more symptoms attributed to illness/better understanding of illnesses’ and higher education were strong facilitators that promoted the uptake of lifestyle behavior change interventions. Stress, depression, lack of social support, transport, and other costs were identified as significant barriers. Similar findings were reported by Kelly et al. [9] in their rapid systematic review, which examined barriers and facilitators to the uptake and maintenance of healthy behaviors by people in mid-life. Across 28 qualitative studies, 11 longitudinal cohort studies, and 46 systematic reviews, the authors identified barriers that included ‘lack of time, access (to transport, facilities, and resources), financial costs, entrenched attitudes and behaviours, restrictions in the physical environment, low socioeconomic status, and lack of knowledge.’ While facilitators included ‘a focus on enjoyment, health benefits including healthy ageing, social support, clear messages, and integration of behaviours into the lifestyle.’

Several theoretical frameworks have been offered to explain health behavior. The socio-ecological model has been widely used to understand interrelations between personal, social and environmental determinants of lifestyle behavior [10-12]. The model is attractive as it incorporates a wide range of influences at multiple levels and variables included in ecological models comprise intrapersonal or personal (biological, psychological), interpersonal/cultural, organizational, physical environment (built, natural), and policy (laws, regulations,).

Singapore is a densely populated city-state in Southeast Asia with a multiracial population of about 5.5 million, comprising Chinese, Malays, Indians, and a smaller proportion of those of other ethnicities [13]. The country has a highly developed free-market economy and had a gross domestic product per capita of about US$60,000 in 2020 [14]. A study of barriers and facilitators of a healthy lifestyle in Singapore is important for the following reasons. Firstly, few studies have examined the barriers and facilitators of a healthy lifestyle in Asian populations. Secondly, the resident life expectancy at birth was 83.9 years in 2020, an increase

from 83.7 years in 2019 and 81.7 years in 2010. This increase heightens the risk of Singapore's population for chronic non-communicable diseases. Thirdly, Singapore has a higher prevalence of diabetes than the global prevalence rate, with nearly one in ten Singaporeans (9.5%) suffering from the disease [15]. At the same time, the total economic cost for working-age patients with diabetes in Singapore, which was USD 787 million in 2010, has been projected to rise to USD 1867 million in 2050 [16], making diabetes a significant public health concern. And lastly, in 2016, the Singapore Ministry of Health declared a "War on Diabetes" to stem the rising prevalence of diabetes. A key component of this campaign is to promote healthy lifestyles among the general population to prevent diabetes.

Therefore, examining factors that influence healthy lifestyles in Singapore against the backdrop of this nationwide campaign is imperative. The findings of such a study can help develop targeted interventions to overcome the barriers and enhance the facilitators to improve the impact of this national campaign. Thus, the purpose of this qualitative study was to provide a comprehensive understanding of barriers and facilitators of healthy lifestyles in the Singapore resident population. We adopted the perspective of the SEM framework to gain a deeper understanding given that personal, cultural (multi-ethnic population), and environmental (backdrop of nationwide campaign and initiatives) factors would substantially influence the lifestyle of the population.

2. Methods

2.1 Study Design and Setting

The data for the current study was part of a more extensive study that examined the knowledge, attitudes, and protective practices towards diabetes among the general public in Singapore. The study comprised a quantitative survey (n=2895) and a qualitative phase (n=30) to explore the barriers and facilitators of a healthy lifestyle in Singapore. The study methodology was published in an earlier article [17].

The participants for the qualitative study were recruited from among those who participated in the quantitative survey and gave permission for recontact to participate in future research studies. Thus, the inclusion criteria comprised Singapore citizens or permanent residents aged 21 years or above, able to speak in either English, Chinese, Malay, or Tamil, and had not been diagnosed by a doctor as having diabetes. In addition, a maximum variation sampling, i.e., equal representation from different gender, age groups, ethnicities, and languages, was used. This allows multiple perspectives to be presented that illustrate the complexity of the phenomenon under study [18].

2.2 Data Collection

As the study period coincided with the COVID-19 pandemic with the attendant social distancing measures, interviews were conducted in person or via the Zoom video-conferencing platform, depending on the participants' preference. Participants were interviewed in venues that afforded privacy so that they could express their views freely. Participants who opted for a Zoom interview were similarly informed that they should ensure a quiet and private setting for the interviews. Each interview was conducted by a trained qualitative interviewer using an interview guide, who was accompanied by a note-taker. The interview guide was informed by existing literature and was modified as the study proceeded to take account of emerging themes (Supplementary File 1). A guided discussion format was used, and participants were encouraged to speak freely about their thoughts and experiences [19]. Interviews were conducted until no new themes emerged, and thematic saturation was met after completing 20 interviews. An additional ten interviews were conducted with native language speakers (Chinese, Malay, and Tamil) to ensure that the perspectives of people who were not well-versed in English and thereby, belonging to unique socio-cultural backgrounds were taken into account.

In all, 30 interviews were conducted from August 2020 to March 2021. 20 of these interviews were in English, four in Chinese, and three each in Malay and Tamil. Each interview lasted for 1-1.5 hours. All the interviews were audio-recorded. Written informed consent was taken from all the participants, and ethical approval for the study was obtained from the relevant institutional review board (National Healthcare Group Domain Specific Review Board; protocol ref:2019/00926).

2.3 Qualitative analysis

The interviews (English and language-specific) were transcribed or translated and transcribed verbatim by a professional transcription firm and checked for accuracy by a study team member. Transcripts were analysed using framework analysis. Framework analysis uses an organized structure of both inductively- and deductively-derived themes [20] to conduct the analysis using a combination of data description and abstraction.

2.4 Data Familiarisation

First, seven researchers (ZYJ, AR, FD, WP, KR, AJ, and MS) familiarised themselves with the transcripts by reading them multiple times. Through this deep engagement with the data and making notes about key themes, the researcher begins to understand major themes in the data. Following the deep reading, initial themes were identified; next, all the authors discussed and combined their themes for comparison. Finally, the researchers frequently met to discuss and resolve disagreements in defining or including themes.

2.5 Framework Identification

The framework was built using a combination of a priori (SEM) and emergent themes [21]. The themes identified in the preceding step were then grouped, to gain a deeper understanding of barriers and facilitators. We completed the framework by identifying the important themes and the conceptual relationship they had to each other.

On reaching a consensus, a codebook was constructed, which contained a detailed description of each code, the inclusion and exclusion criteria, and typical and atypical exemplars to assist with valid and reliable code application.

2.6 Indexing

Each SSI was used as a unit of analysis. Three interviewers (AR, FD, and WP) systematically applied the framework to all the transcripts, after achieving an inter-rater reliability of 0.87 (Kappa (κ) value).

2.7 Mapping and Interpretation

The finalized themes and subthemes were grouped together. We charted the themes with a summary of the main descriptive comments and developed an explanatory account. Representative quotes were selected from the SSI to illustrate key themes and subthemes. These themes and subthemes are represented pictorially in Figure 1.

All analyses were conducted using Nvivo V.11 (QSR International. NVivo V.11).

3. Results

A total of 30 participants (14 females and 16 males) took part in the study. The mean age of participants was 44.7 years (SD=14.7) ranging from 21 to 75 years (Table 1).

Table 1: Socio-demographic profile of participants

Subject ID	Age	Ethnicity	Gender	Language of interview
SS001	25	Chinese	F	English
SS002	24	Chinese	M	English
SS003	39	Malay	F	English
SS004	38	Malay	F	English
SS005	54	Chinese	F	English
SS006	25	Malay	M	English
SS007	34	Malay	M	English
SS008	27	Malay	M	English
SS009	31	Malay	F	English
SS010	22	Chinese	M	English
SS011	53	Chinese	F	English
SS012	55	Malay	F	English
SS013	56	Chinese	M	English

SS014	26	Chinese	F	English
SS015	46	Others	F	English
SS016	38	Chinese	M	English
SS017	24	Others	M	English
SS018	58	Indian	M	English
SS019	52	Indian	M	English
SS020	54	Indian	M	English
SS021	60	Chinese	M	Chinese
SS022	39	Chinese	M	Chinese
SS023	60	Chinese	M	Chinese
SS024	59	Chinese	F	Chinese
SS025	54	Malay	F	Malay
SS026	71	Malay	M	Malay
SS027	65	Malay	F	Malay
SS028	37	Indian	M	Tamil
SS029	61	Indian	F	Tamil
SS030	53	Indian	F	Tamil

Five main themes pertaining to personal, interpersonal, environmental, socio-cultural, and policy-level factors were classified under the two overarching themes of barriers and facilitators of healthy lifestyles. The personal, interpersonal, environmental, and policy-level factors comprised subthemes and are highlighted below. Figure 1 shows the summary of the findings of the themes and sub-themes. Minimally edited verbatims have been included that preserve and highlight the participants' experiences and beliefs. The details of the participants have been provided in brackets as Subject ID/Age/ Gender (**M**ale or **F**emale).

3.1 Barriers to a Healthy Lifestyle

3.1.1 Personal Factors

Personal factors comprised two main subthemes, which explained the barriers to adopting a healthy lifestyle. These included:

Lack of willpower and self-discipline

About one-third of participants mentioned the lack of willpower as an important barrier to maintaining a healthy lifestyle in terms of diet and exercise. They described people as 'being

lazy,' using 'tired,' 'too busy,' and 'work' as an excuse not to partake in physical activity. They also alluded to personal dietary preferences such as liking sweets and desserts and people not having the willpower to resist them. A few of the respondents felt that people were aware of the ill effects of consuming too much sugar, yet they did not have the self-discipline to limit their intake. Respondents similarly felt that in general, people knew about the negative outcomes associated with smoking and alcohol. Yet, they did not quit smoking/ drinking as they did not have the willpower to do it and instead made-up excuses when asked to quit. Respondents also felt that while people may have good intentions and want to adopt a healthy lifestyle, they lacked the willpower to follow through and fell back to unhealthy habits.

"It is your own self-cultivation and self-discipline that has to do with your health. If you don't have good self-discipline and you mess around, what kind of healthy body will you have?" (SS022/39/M)

"It's really hard to change. I have been in contact with a few of them (referring to smokers). One of them stopped smoking for three months. "I stopped smoking." After a few days, he started again. I asked him, 'What do you smoke for?' "Oh, pressure." Pressure is fake." (SS021/60/M)

Lack of knowledge

Participants felt that it was difficult for someone with a chronic health condition to exercise. They also felt that as a person becomes older, they should avoid vigorous exercise as they could injure themselves more easily. Thus, they thought it was advisable for older people and those with chronic health conditions to cut down on their exercise. They did not seem aware that both these groups could exercise safely or substitute high-impact activities for lower-impact ones. Regarding diet, respondents expressed their frustration with contradictory

messages on what was healthy. They felt that food that was once considered healthy was no longer believed to be healthy and vice versa. Thus, they were unsure what should be consumed and what should be avoided. A few respondents identified social media as a significant source of unreliable health information.

"For those who have health conditions, it is very difficult for them to do exercise daily. They are in a life situation where they just cannot take part in a lot of things." (SS030/53/F)

"… especially those messages on social media and WhatsApp about your health. Some people say, "Don't take coconut." Some people say, "Yeah. Coconut is healthy. You take more coconut." So really difficult to judge which is right or which is wrong." (SS019/52/M)

3.1.2 Interpersonal Factors

Negative attitudes and negative influences by family and friends towards a healthy lifestyle

Interpersonal factors were mainly identified as the negative attitudes of family and friends towards a healthy lifestyle or the influence of unhealthy lifestyle practices of friends and family members (such as over-eating) on them. Participants mentioned that friends who made fun of their healthy eating habits were barriers to adopting a healthy lifestyle in the long run. Several participants shared that when they have food with friends, they tended to over-eat or eat food that was not particularly healthy. They felt uncomfortable not eating the food as they were afraid to be perceived as spoilsports if they did not partake in the feasting and drinking.

"I think there's a stigma against healthy food. I know how some of my friends say that eating your salad is girly or whatnot. So maybe eating salad is associated with teenage girls, I guess. I don't know. And I guess maybe if they smoke a stick or drink beer, it is more associated with masculine ideals. So maybe if you don't follow the party or whatnot, you may be viewed as an

outlier or something. So yeah. I would say there is some social stigma tied to certain types of food or lifestyle, I guess." (SS010/22/M)

"...for example, go out with friends and drink milk tea (referring to bubble milk tea, a sweet tea with tapioca balls that is very popular in Singapore) together, and then he drinks a cup and buys you a cup. Will you not drink it? Sometimes, it's not very polite to say no to your friends." (SS022/39/M)

3.1.3 Environmental factors

As most of the interviews were conducted during the COVID-19 pandemic when outdoors activity was restricted, it was not surprising that participants mentioned the pandemic as a significant barrier to physical activity. The fear of infection, as well as safe distancing measures and other restrictions hindered participants from performing outdoor activities and those conducted in gyms or enclosed spaces. The other sub-theme that emerged, pertained to situational factors such as conflicting demands leading to time constraints and the low cost and ready availability of fast food that were perceived to be barriers to adopting a healthy lifestyle.

COVID-19 pandemic

All the participants mentioned the impact of the COVID-19 pandemic on their lifestyles and expressed their fears and frustrations. For example, participants talked about how the social distancing regulations, closure of indoor gyms and training spaces, and the need to mask up (even in outdoor spaces in Singapore) were significant barriers to exercising in Singapore. Moreover, putting a mask on for most of the day in Singapore's hot and humid weather left them feeling tired, irritable, and reluctant to exercise.

“Actually, before COVID, my friends and I played soccer on a weekly basis, every Sunday. Yeah. But because of COVID, then we stopped completely.” (SS008/27/M)

“And looking at the number of cases (Covid cases), they went up higher. So it’s a deterrence to exercising.” (SS022/39/M)

Situational factors

The most discussed barrier was time constraints associated with competing priorities such as employment, household chores, and looking after children or older parents. However, other factors such as financial constraints and limited access to healthy food were also reported to impact healthy lifestyle behaviors.

Participants mentioned that healthy food was both expensive and not readily available. They acknowledged that fast food was the most convenient food, and while they knew that consuming a diet rich in calories was associated with being overweight, they were unable to avoid it. The easy availability of fast food at all hours and food stalls that stayed open even during the night in Singapore also encouraged poor food habits. Interestingly, food delivery was associated with an unhealthy lifestyle. Participants felt that food delivery led to people ordering more due to its convenience and easy availability, in addition to that it also led to them eating at odd hours.

While most participants acknowledged that Singapore had several parks and exercise areas that were conducive to physical activity, three of the respondents had concerns about the safety of these facilities. These included dimly lit parks that made it difficult to walk in the night, sharing of the same path by pedestrians, cyclists, and children and teenagers who tended to run or skate, thus increasing the risk of accidents among older adults. In addition, two participants

highlighted the lack of good cycling tracks in Singapore, which does not encourage a cycling culture unlike Denmark or the Netherlands.

"Because sometimes, frankly speaking, children are young, and it is hard for you to have any free time for yourself." (SS024/59/F)

"I think maybe the fact that healthy food is quite expensive in Singapore. So I guess food, in general, can be quite affordable if you go hawker center or whatnot. But then they are generally not very healthy, so it can be quite troublesome for some people to cook healthier food." (SS010/22/M)

"Now you can even use your phone to just order, and they will deliver it directly to your house. Ordering food or eating outside food has become so easy that it has become a part of their lifestyle. So they don't give much consideration to the food itself, and a healthy lifestyle is lost." (SS030/53/F)

Cultural factors

Several cultural factors emerged as barriers to the adoption of healthy lifestyles. Given the multi-ethnic nature of Singapore, barriers pertaining to cultural factors were identified both by people belonging to that ethnocultural group and others. These included:

The cultural importance of traditional food

Participants acknowledged that cooking and eating traditional food was an important ritual in Singapore. However, about one-third of the participants felt that Indian and Malay food tended to be oily and calorie-rich. They also acknowledged that these types of food appealed to people's tastes, and they ended up overeating it. In addition, they felt that desserts unique to these cultures were similarly sweet and not healthy. Coconut milk in traditional food preparations was similarly identified as an unhealthy but a necessary ingredient. Some had

also commented, that those belonging to the Chinese ethnicity liked to eat pork and were not willing to switch to healthier meat alternatives (such as white meat). They also acknowledged that Chinese cuisine could be oily as there are many dishes that are deep-fried.

"In fact, Singapore's food is not only western food, but also Malay food, Indian food, and Chinese food. It doesn't contain as much oil as Chinese cuisine in China. Chinese food has a lot of oil. The Chinese food here, oil and salt, will not be so overused, but it will have a lot of fried things. And your Indian food and Malay food, I believe it will have a lot of sugar, especially Malay food." (SS022/39/M)

Customs and Festivals

Participants also talked about food habits of specific ethnic groups, such as eating dinner late in the night and close to bedtime which they perceived as unhealthy. They also acknowledged that festive periods were not conducive to maintaining a healthy lifestyle as it was all about meeting friends and families and eating. So one tended to over-eat during such periods.

"As far as we Chinese are concerned, if we talk about the Chinese New year, it may be that everyone eats more…" (SS021/60/M)

Language barriers

People from the minority ethnicities expressed their reluctance to participate in community exercises as they felt that they would not be able to understand the instructions as these tend to be conducted in a language that they are not conversant in.

"…that's why I just hate to go to some of these community activities. They are all in English, and I won't understand. Most or all are in English only, so I feel a little uncomfortable because of that." (SS030/50/F)

3.1.4 Policy related factors

Policy-related barriers did not emerge very strongly in this group of participants. However, a few participants expressed their frustration with the reluctance of the government to impose a sugar tax. They felt that sugar caused significant harm to a person's health, but it was not something that could be taxed. They mentioned the 'bubble tea fad' in Singapore, which led to the opening of several shops across the country that sold sweet and calorie-rich drinks. The existing policies could not limit such shops; the government they felt could only advocate and educate people about the potential harms of such food.

"I don't think it's realistic. Because, for example, the government tells people not to smoke, then they increase the tax on cigarettes. Don't drink, and they will add a high tax to wine. But it is impossible to add a high tax on sugar because sugar is a necessity in life. Unlike tobacco and wine, it is not a luxury but a part of the diet. Many people in their daily life use sugar. So you can't, the government can't say, add a high tax to milk tea shops. So I think from the government's point of view that they can't do many things. They can just advocate." (SS022/39/M)

3.2 Facilitators of a Healthy Lifestyle

3.2.1 Personal and Interpersonal factors

Most of the facilitators highlighted by the participants in these two themes were the opposite of those mentioned as barriers, although the absence of a barrier was not necessarily a facilitator. For example, personal facilitators highlighted by the majority of participants were ‘willpower and motivation.’ Participants talked about how willpower was necessary to exercise regularly and eat healthy food. They also felt that if people knew the impact of a healthy lifestyle on long-term outcomes, they would commit to them. One-third of participants also acknowledged that people with health conditions should continue to exercise and maintain a healthy diet as it can prevent secondary complications. They also highlighted the important role of friends and family members in encouraging and supporting a healthy lifestyle, which helped the participants maintain it.

3.2.2 Organisational/ Institutional factors

Workplace initiated health promotion interventions

The workplace emerged as a significant facilitator of a healthy lifestyle. More than half of the participants who were employed mentioned various workplace initiatives that had helped them to become more physically active. This was mainly through workplace wellbeing initiatives such as distribution of fruits, subsidized fruit bazaars at the place of work, educational sessions on diet and its impact on wellbeing, and group exercise classes like Zumba or Yoga. Many workplaces continued these initiatives even during the pandemic by leveraging on Zoom and other platforms.

“But my workplace, I would say they are trying to endorse the whole healthy lifestyle thing. So we do have things like a monthly fruits giveaway. So every single month we’ll get different kinds of fruit and then staff will explain to us what’s the benefit of eating food, or fruit rather. We have staff exercise sessions where you can sign up for yoga or gym sessions or go for a walk.” (SS009/31/F)

Influence of healthcare and other professionals

Several participants talked about adopting a healthier lifestyle after their healthcare provider (usually a doctor or dietician) advised them about healthy eating or physical activity. They also spoke of informative media programs that encouraged the adoption of a healthy lifestyle.

"Polyclinics (primary care clinics) or I think if I didn't recall wrongly-- I can't really remember. Is it one of the hospitals or polyclinics my parents visited? They actually have a nutritionist who tells you what to eat." (SS016/38M)

"White meat, the doctor's advice is to eat more white meat instead of red meat because red meat is not good for cholesterol." (SS022/39/M)

3.2.3 Environmental factors

Role of Technology as a facilitator of a healthy lifestyle

All the participants highlighted the role of technology as a facilitator of a healthy lifestyle. However, their understanding of technology was varied. Any source of information like Television, Radio, Internet search engines, channels like YouTube, social media sites, and Apps (mobile applications) was described as technology. The role of technology was mainly seen to be threefold. Firstly as a source of information on diet and exercises (information which could be conveyed via articles, programmes/ shows, talks, etc.), secondly as a guide for doing or aiding training, cooking low-calorie food, etc., and lastly for tracking and monitoring their physical activity, heart rate, sleep, as well as food consumption. Some of the participants alluded to Apps that sent them reminders to breathe deeply, meditate, and walk as helpful.

"App for cycling that's called Strava. I think that's just the only healthy fitness app that I have. So they will keep track of your heartbeat, the distance from one point to another, and the speed of cycling." (SS007/34/M)

"It's MyFitnessPal. Well, it tracks my calorie intake for the day. And it's quite specific. It's quite good. But I think they cannot detect some of the local foods. But other than that, they can track basically whatever that goes in your mouth, yeah, whatever you consume. And you can put in your exercises for the day. So, yes, it will help you calculate your goals, like how many KG you want to lose in a month also, which is quite good." (SS008/24/M)

3.2.4 Policy-related factors

The participants highlighted several policy-related facilitators. These included:

Built spaces and exercise stations

More than half of the participants talked about the availability of neighbourhood parks which provided a safe and convenient place to exercise. Participants mentioned that walks were often organised in their neighbourhoods and that volunteers would encourage them to join in these activities. They were also aware that several activities were conducted in these spaces that one could join at no cost. Some also felt that such group activities motivated them, and they enjoyed doing these more than doing exercises by themselves. Participants also mentioned that there are public swimming pools that one could use and government-run gyms where one could access high-quality equipment at a minimal cost. Participants acknowledged that the government was constantly upgrading parks and gyms, and they could now easily access parks and exercise corners.

"Walking, our government is good, gave us many parks and so many connectors (scenic roads connecting parks where pedestrians can walk). You cannot say that there are no facilities." (SS024/59/F)

"Sometimes they do come and call me to join, like what's that called, social service Community volunteers. When we exercise with other people in a group, it gives us a sort of motivation." (SS030/53/F)

Inclusion of physical education as a core curriculum in school

About a quarter of the participants mentioned that including physical education in schools and getting children to exercise regularly as part of the school curriculum encouraged incorporating exercise into their lifestyle. They also shared that even institutes of tertiary education offered an excellent array of exercise classes/ options, which encouraged the students to continue exercising.

"The education system right now really talks about mental health and physical health. Even their physical education is different. So I think exposure to that will be one of the factors that will enable them to live a healthy lifestyle." (SS009/31/F)

"So when I went to XXX Poly [polytechnic name], I started Muay Thai. And even the gym was affiliated with XXX [polytechnic name]. So I've been with this gym since the dawn of time. This is my first gym, and I've been with them all the way [laughter]." (SS006/25/F)

Figure 1 Barriers and Facilitators of adopting a healthy lifestyle

4. Discussion

This article explored the barriers and facilitators of a healthy lifestyle perceived and experienced by a multi-ethnic sample of adults in Singapore. Using a framework analysis approach that comprised two major components: creating an analytic framework and applying this analytic framework, we leveraged the SEM [12] model, to gain a deeper understanding of the barriers and facilitators of a healthy lifestyle. The discussion focuses on key themes that lend well to intervention or were unique to this study.

At the personal level, lack of willpower emerged as a key barrier, while being motivated and having the resolve to exercise or not eat sweet or calorie-rich food, despite the challenges, was identified as a facilitator. Willpower, defined as the capacity to exert self-control, has emerged

in several studies as a barrier to healthy eating [22,23] and physical activity [24]. In Tsukayama et al. [25]'s prospective longitudinal study, they found that more self-controlled children were less likely to become overweight as they entered adolescence. Cognitive and behavioral interventions have been developed to promote self-regulation [26] and overcome this barrier. For example, a study among women aged 30-50 years showed that a brief intervention combining information with a self-regulation technique (integrating mental contrasting with implementation intentions) led to the maintenance of high consumption of fruits and vegetables 24 months after the intervention. In contrast, the information-only intervention group returned to baseline consumption of fruits and vegetables [27].

Environmental factors that emerged as barriers and facilitators were unique to this study. The study period coincided with the COVID-19 pandemic, and all the participants mentioned the pandemic as a barrier to the adoption of a healthy lifestyle. While some participants spoke about the importance of their own 'willpower' in maintaining their exercise regimen during the pandemic, they acknowledged the challenge posed by the pandemic. Other studies have similarly reported dramatic lifestyle changes in terms of reducing physical activity with increased sedentary behaviors and reduced physical activity during the Covid-19 pandemic [28, 29]. These unhealthy lifestyle behaviors observed in the pandemic period can potentially lead to the persistence of these poor lifestyle habits and the development of chronic diseases. There is a need to develop and implement interventions to maintain a healthy lifestyle under quarantine and beyond it, paying particular attention to high-risk groups such as individuals with pre-existing physical health problems and older adults.

Several cultural factors emerged as barriers to adopting a healthy lifestyle in this study. Prevalence of diabetes varies among Chinese, Malay, and Indian ethnicities [15], and it is often ascribed to dietary differences, especially in the popular media. It was interesting to note that while some participants of Indian and Malay ethnicities said that their food choices and food

preparation might be high in calories, many Chinese participants also commented that traditional Malay or Indian food was too sweet or oily. However, most of our participants acknowledged cross-cultural eating and said they preferred deep-fried or sweet food. They also felt that they ended up eating more of it than low-fat options that were often not spicy or tasty. All the major festivals celebrated in Singapore were identified as periods where people choose not to count calories and enjoy feasting with friends and families, highlighting the importance of traditional food during social gatherings and religious or traditional celebrations. A study on South Asian immigrants in Australia identified a similar theme where participants felt that food was a central theme of social gatherings and indicated their preference for traditional food in these settings [30].

Workplace and technology emerged as significant environmental facilitators. The Health Promotion Board, Singapore, has spearheaded workplace initiatives. They identified workplaces as a key setting as most adult Singapore residents spend most of their day at work. The focus areas include obesity prevention and management and chronic disease management. They work proactively with companies to support them with the necessary tools to ensure a health-promoting workplace. These initiatives have resulted in many companies providing workplace talks on physical and mental health, organising group physical activities and providing healthy food alternatives in canteens, subsidised fruit sales, and distributing fruits and healthy snacks to staff [31]. Technology has been classified as a component of the physical environment's artificial elements [32]. Given the focus on developing Singapore as a 'smart nation' to leverage technology and implement it nationally, the widespread interest and adoption of technology for a healthy lifestyle was not surprising. With the rapid technological advances and integration of smartphones with wearable devices that are capable of assessing physical activity, sedentary behavior, heart rate and intensity levels of physical activity [33],

we found that people in general and especially young people preferred wearables as facilitators of a healthy lifestyle.

Furthermore, technological advancement has resulted in better identification of previously non-identifiable physical activity (e.g., stair climbing, outdoor cycling) which our participants mentioned as particularly appealing as they catered to their lifestyle. Participants also alluded to the persuasive technology that was often incorporated within the wearables. The ability to send their achievements (hours exercised, distance covered, etc.) to online communities or friends with whom they could compete, stay accountable or get encouragement for their achievements was seen as a facilitator by some participants. At the same time, others mentioned the reminders to pause and take deep breaths as being useful in the middle of stress-filled days. Persuasive technology is defined as technology that is designed to change individuals' attitudes or behaviors through persuasion and social influence, but not through coercion [34]. Technological advances can be used to nudge individuals to engage in more physical activity. This is done by capturing the data and comparing it with historical data; tracking improvement over time; linking data to social media; and sending encouraging messages to the wearer, such as asking them to move more or taking some time for deep breathing or mindfulness. While these are exciting and transformative developments that facilitate the adoption of a healthy lifestyle, little data is available concerning how successful these apps or wearables are at enabling users to lose weight or get fit over time. Research into the effectiveness of many of these technologies is still in its infancy.

Policy-driven changes to the built environment and education curriculum were identified by participants as facilitators of the adoption of a healthy lifestyle. Our study highlights the importance of positioning health promotion in city planning and developing 'healthy built environments.' Surprisingly, participants did not mention the 'utilitarian walking' encouraged in Singapore due to greater land-use mixes that ensure easy accessibility to various locations

in the neighbourhood like shops, food courts, and primary care services. Instead, they focused mainly on recreational walking enabled by small open places like playgrounds and large parks within walking distance of the residential neighbourhoods. Many pointed out that with other walkability features such as safe sidewalks, covered pathways, and easy accessibility, there was no excuse not to exercise in Singapore. Several other studies in Singapore have similarly established that physical activity levels are closely associated with the built environment characteristics [35,36].

The incorporation of physical education classes in the curriculum meets several important objectives. First, it ensures that students participate in appropriate amounts of physical activity during lessons. Secondly, they become equipped with the knowledge and skills to be physically active throughout their life [37]. Several participants referred to the need to incorporate physical activities in the routine right from childhood as they felt that children who learn these skills would use them lifelong. And that it is more difficult to convince an older adult to do physical activities, especially if they have not done them before. However, schools should consider providing a diverse range of physical activity experiences so that the needs and interests of all children are met. Schools should also consider incorporating healthy eating practices as part of their curriculum to further reduce the risk of childhood obesity and promote lifelong healthy nutritional practices [38].

There are some limitations to our study. Since the study was planned before the COVID-19 pandemic, in the early part of the study, the team did not have specific questions or probes that examined factors that could be related to the pandemic. The pandemic may have also limited those who were not technologically savvy or worried about the impact of the infection through interviews, from participating in the study. While the study allowed the participants to define what a healthy lifestyle meant to them, the discussion on barriers and facilitators centered mainly around physical activity and nutrition. There was limited discussion around other

aspects of a healthy lifestyle like using tobacco, alcohol, sleep, and mental health. The strengths of our study include a good representation of people across ethnic groups and languages in a multi-ethnic population. The use of one-to-one interviews that led to frank discussions on barriers and facilitators and the inclusion of data from 30 interviews ensured thematic saturation. The qualitative researchers involved in this study came from different disciplines, thus providing a transdisciplinary understanding of the phenomenon under study. The study results triangulate well with those of other studies examining barriers and facilitators of healthy lifestyles.

5. Conclusions

The results of our study offer important insights into understanding the barriers and facilitators to the adoption of a healthy lifestyle among people in Singapore. Barriers and facilitators have a complex interplay within the SEM framework. Our findings illustrate how internal willpower and self-regulation can act as either a barriers or a facilitator to adopting a healthy lifestyle at the personal level. At the interpersonal level, influence from family and peers can similarly act as either barriers or facilitators. Technological devices for monitoring activities and diet emerged as significant facilitators that can be further leveraged to improve the health of populations. The workplace environment and policy set the foundations for the availability of health-promoting options and play a crucial role in ensuring the adoption of a healthy lifestyle. Most of the barriers identified are amenable to interventions. Incorporation of educational material with motivational techniques or short intervention to improve self-regulation and delivered by health care professions, or the evaluation of nudging using technology as a way to promote healthy habits in the youth should be explored in future studies. The SEM framework supports the evidence that an individual supported by both environment and policy can make healthy lifestyle choices and prevent or delay the onset of chronic medical conditions and age successfully.

Supporting Information

S1 File: Semi-structured interview guide

Acknowledgement: This study is supported by the Singapore Ministry of Health's National Medical Research Council under its Health Services Research Grant (NMRC/HSRG/0085/2018).

References

1. Centers for Disease Control and Prevention (CDC). Ten great public health achievements-United States, 2001-2010. MMWR Morb Mortal Wkly Rep. 2011;60(19): 619–23.
2. McKeown RE. The Epidemiologic Transition: Changing Patterns of Mortality and Population Dynamics. Am J Lifestyle Med. 2009;3(1 Suppl):19S–26S. doi: 10.1177/1559827609335350.
3. Elwyn G, Laitner S, Coulter A, Walker E, Watson P, Thomson R. Implementing shared decision making in the NHS. BMJ. 2010;341:c5146.
4. Piepoli, MF, Hoes AW, Agewall S, Albus C, Brotons C, Catapano A, et al; ESC Scientific Document Group 2016 European guidelines on cardiovascular disease prevention in clinical practice: the sixth joint task force of the European Society of Cardiology and other societies on cardiovascular disease prevention in clinical practice (constituted by representatives of 10 societies and by invited experts). Developed with the special contribution of the European Association for Cardiovascular Prevention & Rehabilitation (EACPR). Eur Heart J. 2016;37(29):2315–81.
5. Lindström J, Ilanne-Parikka P, Peltonen M, Aunola S, Eriksson JG, Hemiö K, et al. Sustained reduction in the incidence of type 2 diabetes by lifestyle intervention: follow-up of the Finnish diabetes prevention study. Lancet. 2006;368(9548):1673–9.
6. World Health Organisation. What is a healthy lifestyle? EUR/ICP/LVNG 01 07 02. E66134. WHO Regional Office for Europe, Copenhagen, 1999.
7. Pender NJ, Murdaugh CL, Parsons MA. Health Promotion in Nursing Practice. 5th ed. Upper Saddle River, NJ: Prentice-Hall, 2006.
8. Murray J, Craigs CL, Hill KM, Honey S, House A. A systematic review of patient reported factors associated with uptake and completion of cardiovascular lifestyle

behaviour change. BMC Cardiovasc Disord. 2012;12:120. https://doi.org/10.1186/1471-2261-12-120.

9. Kelly S, Martin S, Kuhn I, Cowan A, Brayne C, Lafortune L. Barriers and Facilitators to the Uptake and Maintenance of Healthy Behaviours by People at Mid-Life: A Rapid Systematic Review. PLoS ONE. 2016;1(1):e0145074. doi:10.1371/journal.pone.0145074.
10. Hurowitz JC. Toward a social policy for health. N Engl J Med. 1993;329:130–3.
11. Hill JO, Galloway JM, Goley A, Marrero DG, Minners R, Montgomery B, et al. Scientific statement: Socioecological determinants of prediabetes and type 2 diabetes. Diabetes Care. 2013;36:2430–9.
12. McLeroy KR, Bibeau D, Steckler A, Glanz K. An ecological perspective on health promotion programs. Health Educ Q. 1988;15:351–37.
13. Department of Statistics, Singapore. Census of population, population trends, 2021. Available: https://www.singstat.gov.sg/-/media/files/publications/population/population2018.pdf [Accessed 5 March 2022]
14. World Bank. Data: GDP per capita (current US$) [Last assessed 5 March 2022]. Available from http://data.worldbank.org/indicator/NY.GDP.PCAP.CD.
15. Ministry of Health and Health Promotion Board, Singapore Epidemiology & Disease Control Division and Policy, Research & Surveillance Group. National Population Health Survey 2020. Available online at https://www.moh.gov.sg/docs/librariesprovider5/default-document-library/nphs-2020-survey-report.pdf Last accessed on 4th May 2022.
16. Nanditha A, Ma RC, Ramachandran A, Snehalatha C, Chan JC, Chia KS, et al. Diabetes in Asia and the Pacific: Implications for the Global Epidemic. Diabetes Care. 2016;39: 472–85.

17. AshaRani PV, Abdin E, Kumarasan R, Siva Kumar FD, Shafie S, Jeyagurunathan A, et al. Study protocol for a nationwide Knowledge, Attitudes and Practices (KAP) survey on diabetes in Singapore's general population. BMJ Open. 2020;10(6):e037125.
18. Sandelowski M. Focus on quantitative methods: Sample sizes, in qualitative research. Res Nurs Health. 1995;18:179–83.
19. Kvale S, Brinkmann S. Interviews. In: Learning the Craft of Qualitative Research Interviewing. Sage, 2009.
20. Goldsmith LJ. Using Framework Analysis in Applied Qualitative Research. Qual Rep. 2021;26(6):2061–76.
21. Ritchie J, Spencer L. Qualitative data analysis for applied policy research. In A. Bryman & R. Burgess (Eds.), Analyzing qualitative data (pp. 305–329). Routledge, 1994.
22. McMorrow L, Ludbrook A, Macdiarmid JI, Olajide D. Perceived barriers towards healthy eating and their association with fruit and vegetable consumption. J Public Health. 2017;39:330–8.
23. Wongprawmas R, Sogari G, Menozzi D, Mora C. Strategies to Promote Healthy Eating Among University Students: A Qualitative Study Using the Nominal Group Technique. Front Nutr. 2022;9:821016.
24. Justine M, Azizan A, Hassan V, Salleh Z, Manaf H. Barriers to participation in physical activity and exercise among middle-aged and elderly individuals. Singapore Med J. 2013; 54(10):581–6.
25. Tsukayama E, Toomey SL, Faith MS, Duckworth AL. Self-control as a protective factor against overweight status in the transition from childhood to adolescence. Arch Pediatr Adolesc Med. 2010;164(7):631–5.

26. Smith T, Panfil K, Bailey C, Kirkpatrick K. Cognitive and behavioral training interventions to promote self-control. J Exp Psychol Anim Learn Cogn. 2019;45(3):259–79.

27. Stadler G, Oettingen G, Gollwitzer PM. Intervention effects of information and self-regulation on eating fruits and vegetables over two years. Health Psychol. 2010;29(3): 274–83.

28. Giuntella O, Hyde K, Saccardo S, Sadoff S. Lifestyle and mental health disruptions during COVID-19. Proc Natl Acad Sci USA. 2021;118(9):e2016632118. doi: 10.1073/pnas.2016632118.

29. Caroppo E, Mazza M, Sannella A, Marano G, Avallone C, Claro AE, et al. Will nothing be the same again?: Changes in lifestyle during COVID-19 pandemic and consequences on mental health. Int J Environ Res Public Health. 2021;18(16):8433.

30. Nisar M, Khan A, Kolbe-Alexander TL. 'Cost, culture and circumstances': Barriers and enablers of health behaviours in South Asian immigrants of Australia. Health Soc Care Community. 2022; Feb 18. doi: 10.1111/hsc.13759.

31. Health Promotion Board. Workplace 2020. Available online at https://www.hpb.gov.sg/workplace

32. Gadais T, Boulanger M, Trudeau F, Rivard MC. Environments favorable to healthy lifestyles: A systematic review of initiatives in Canada. J Sport Health Sci. 2018;(1):7–18.

33. Graham DJ, Hipp JA. Emerging technologies to promote and evaluate physical activity: Cutting-edge research and future directions. Front Public Health. 2014;2:66.

34. Dominic D, Hounkponou F, Doh R, Ansong E, Brighter A. Promoting physical activity through persuasive technology. Int J Invent Eng Sci. 2013;2:16–22.

35. Tao Y, Zhang W, Gou Z, Jiang B, Qi Y. Planning Walkable Neighborhoods for "Aging in Place": Lessons from Five Aging-Friendly Districts in Singapore. Sustainability. 2021; 13(4):1742.

36. Song S, Yap W, Hou Y, Yuen B. Neighbourhood built environment, physical activity, and physical health among older adults in Singapore: A simultaneous equations approach. J Transp Health. 2020;18:100881.

37. Simons-Morton BG. Implementing health-related physical education. In Pate RR and Hohn RC. (eds), Health and Fitness Through Physical Education. Human Kinetics, Champaign, IL, 1994;137–46.

38. Swindle T, Rutledge JM, Selig JP, Painter J, Zhang D, Martin J, et al. Obesity prevention practices in early care and education settings: an adaptive implementation trial. Implementation Sci. 2022;17:25.

 Click here to access/download;Figure;Figure 1.jpg

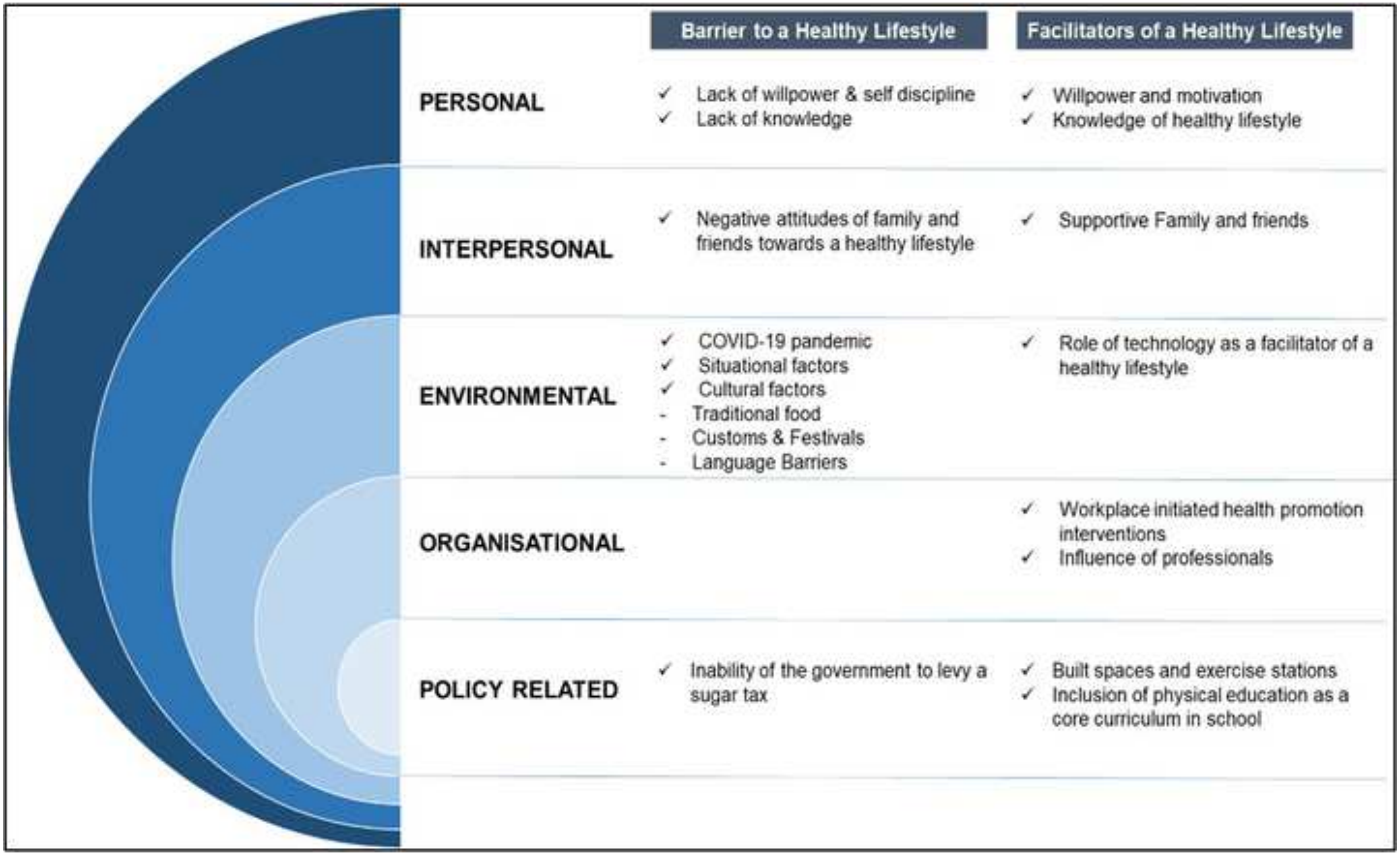

Click here to access/download
Supporting Information
S1 Interview guide.docx
