# Supplementary material for: Barriers and facilitators for adopting a healthy lifestyle in a multi-ethnic population: A qualitative study
Source: PLoS One. 2022 Nov 2;17(11):e0277106. doi: 10.1371/journal.pone.0277106 (PMC9629631; doi:10.1371/journal.pone.0277106)
Supplement: Attachment — Submitted filename: PONE-D-22-13181_R1_Ed.pdf [file pone.0277106.s005.pdf]

PLOS ONE

Barriers and Facilitators for adopting a healthy lifestyle in a multi-ethnic population: A qualitative study

--Manuscript Draft--

**Manuscript Number:**	PONE-D-22-13181R1
**Article Type:**	Research Article
**Full Title:**	Barriers and Facilitators for adopting a healthy lifestyle in a multi-ethnic population: A qualitative study
**Short Title:**	Barriers and Facilitators of a Healthy Lifestyle
**Corresponding Author:**	Mythily Subramaniam<br>Institute of Mental Health<br>Singapore, SINGAPORE
**Keywords:**	healthy lifestyle; willpower; family; Technology; qualitative
**Abstract:**	A healthy lifestyle is defined as 'a way of living that lowers the risk of being seriously ill or dying early.' Although barriers and facilitators of healthy lifestyles have been well-studied among populations like those with chronic non-communicable diseases, adolescents, and older adults in Asia, less information is available on barriers and facilitators perceived by the general adult population. The current study leveraged the socio-ecological model to gain a deeper understanding of the barriers and facilitators of a healthy lifestyle in a sample of Singapore residents using a qualitative methodology. In all 30 semi-structured interviews were conducted in English and other local languages from August 2020 to March 2021. Transcripts were analysed using framework analysis. Five main themes pertaining to personal, interpersonal, environmental, socio-cultural, and policy-level factors were classified under the two overarching categories of barriers and facilitators of healthy lifestyles. The results of this study offer important insights into understanding the barriers and facilitators to the adoption of a healthy lifestyle among people in Singapore. Our findings illustrate the complex interplay between individuals, social relationship, environment, and policy that can act as either a barrier or a facilitator to adopting a healthy lifestyle.
**Order of Authors:**	Mythily Subramaniam
	Fiona Devi
	P.V. AshaRani 1
	Yunjue Zhang
	Peizhi1
	Anitha Jeyagurunathan
	Kumarasan Roystonn
	Janhavi Ajit Vaingankar
	Siow Ann Chong
**Opposed Reviewers:**	
**Response to Reviewers:**	27 August 2022<br>Eliana Carraça<br>Academic Editor<br>PLOS ONE<br><br>Ref: PONE-D-22-13181<br>Barriers and Facilitators for adopting a healthy lifestyle in a multi-ethnic population: A qualitative study<br><br>Dear Dr. Carraca<br>We would like to thank you and the reviewers for your constructive review. We have addressed the points raised by the reviewers in the revised manuscript in as tracked

Powered by Editorial Manager® and ProduXion Manager® from Aries Systems Corporation

changes. Our replies to their comments are attached and highlighted in bold for easy reference. The major comments on the pdf have been addressed separately as we are not sure which reviewer has commented on them. The minor comments have been addressed directly in the text as tracked changes.
Academic Editor
The topic of this paper is of interest to the Plos One scientific community. Adherence to healthy lifestyle behaviours remains a challenge, thus exploring which factors might facilitate or hinder the adoption of these behaviours is critical to improve intervention's design and efficacy. Still, the current paper does not show the novelty of this study. This should be made totally clear in all sections of the paper, but most importantly in the introduction and discussion sections. Authors are also advised to be more specific and support all affirmations in the literature.
We have added relevant references and clarified the novel aspects of this study.

The methodology should be clearer, especially regarding the process of development of the instrument and how the framework analysis was conducted.
The methodology has been substantially revised as suggested by the reviewers.
The impact of COVID on recruitment and on this study should be discussed, as these results could be different in a normal, non-COVID, situation. Being the first study on the Singapore population, this should be interpreted with caution and unlikely generalisable. Therefore, avoid using terms that imply that your findings are comprehensive and generalisable to all Singapore population within the same age range.
We have avoided the use of terms that suggest that the findings are comprehensive and generalizable.

Grammatical errors should be carefully revised.
Grammatical errors have been revised.
Journal Requirements
When submitting your revision, we need you to address these additional requirements.
1. Please ensure that your manuscript meets PLOS ONE's style requirements, including those for file naming. The PLOS ONE style templates can be found at
2. Thank you for stating the following in the Acknowledgments Section of your manuscript:
"This study is supported by the Singapore Ministry of Health's National Medical Research Council under its Health Services Research Grant (NMRC/HSRG/0085/2018)."
We note that you have provided additional information within the Acknowledgements Section that is not currently declared in your Funding Statement. Please note that funding information should not appear in the Acknowledgments section or other areas of your manuscript. We will only publish funding information present in the Funding Statement section of the online submission form.
Please remove any funding-related text from the manuscript and let us know how you would like to update your Funding Statement. Currently, your Funding Statement reads as follows:
"SAC received the funding
NMRC/HSRG/0085/2018
This study is supported by the Singapore Ministry of Health's National Medical Research Council under its Health Services Research Grant
The funders had no role in study design, data collection and analysis, decision to publish, or preparation of the manuscript."
Please include your amended statements within your cover letter; we will change the online submission form on your behalf.
We would like to retain the statement as it is in the online submission form. We have removed the statement in the revised manuscript.
3. In your Data Availability statement, you have not specified where the minimal data set underlying the results described in your manuscript can be found. PLOS defines a study's minimal data set as the underlying data used to reach the conclusions drawn in the manuscript and any additional data required to replicate the reported study findings in their entirety. All PLOS journals require that the minimal data set be made fully available. For more information about our data policy, please see.
Upon re-submitting your revised manuscript, please upload your study's minimal underlying data set as either Supporting Information files or to a stable, public

repository and include the relevant URLs, DOIs, or accession numbers within your revised cover letter. For a list of acceptable repositories, please see. Any potentially identifying patient information must be fully anonymized.
Important: If there are ethical or legal restrictions to sharing your data publicly, please explain these restrictions in detail. Please see our guidelines for more information on what we consider unacceptable restrictions to publicly sharing data: https://imsva91-ctp.trendmicro.com:443/wis/clicktime/v1/query?url=http%3a%2f%2fjournals.plos.org%2fplosone%2fs%2fdata%2davailability%23loc%2dunacceptable%2ddata%2daccess%2drestrictions&umid=C1935020-E531-7305-AE14-9D67384FA132&auth=6e3fe59570831a389716849e93b5d483c90c3fe4-a54b28680ec209732c5562257d5ed9d73bc73984. Note that it is not acceptable for the authors to be the sole named individuals responsible for ensuring data access.
We will update your Data Availability statement to reflect the information you provide in your cover letter.
Revised data availability statement: The data underlying the results presented in the study are available from the first author Dr Mythily Subramaniam (Mythily@imh.com.sg)
Reviewer: 1

1) As the authors note, many studies have been published describing barriers and facilitators to healthy lifestyle practices. While this study is novel in its focus on Singapore, the results do not seem to differ much at all from the large body of work on this topic. It would be useful in the discussion to frame how the findings of this study compare to other similar studies. Are there barriers and facilitators specific to adults in Singapore that could be targeting in a healthy lifestyle intervention?
We would like to thank the reviewer for this suggestion. We have revised our introduction to address how the study is different from others that have been carried out in other countries.

2) Authors make the claim that this study provides a "comprehensive" understanding of barriers and facilitators (pg 5). I am not sure this claim can be made with a fairly small sample (n=30). The paper can still bring forward important findings without being comprehensive.
We have removed the word ‘comprehensive’.

3) Pg 8: spell out "SSI" on first use
We apologise for the error and have added the full form as suggested by the reviewer.

4) Pg 8, table 1: Given the important of education level and adoption of healthy lifestyle habits (as the authors mention in the introduction), it's surprising that education is not included in the demographics. It would also be interesting to see how barriers and facilitators differed among those with varying levels of education.
We have included the educational backgrounds of the participants in the revised table.

5) Similarly to the comment above, it would have been nice to see a segmentation of the data by demographics. For example, are barriers and facilitators different for men and women? Older and younger people? By ethnicity and/or language?
We are unable to do such an analysis as the study design was set up to get a good representation across the groups but did not set out to examine differences across the groups. The sample size does not lend to such a nuanced segmentation and we apologise for that.

6) pg 14: What is meant by "hawker center" in the quote? Provide definition or use a different quote.
We have explained what is meant by a ‘hawker center’. We think it is important to retain this quote as it highlights the availability of affordable food which is often not healthy.

7) pg 16: What is meant by "community exercises"? Is this physical activity opportunities in the community?
We have clarified the term by changing it to community-based group exercise programs for clarity.

8) Informed consent was collected from all participants. Was the study protocol

reviewed and approved by an institutional review board?
Yes, we have clearly stated just below the statement on consent that - ethical approval for the study was obtained from the relevant institutional review board (National Healthcare Group Domain Specific Review Board; protocol ref:2019/00926).

9) In the introduction, the authors state that this paper will contribute to developing interventions to promote healthy lifestyles among people in Singapore, where diabetes and other chronic conditions are on the rise. I would therefore expect to see explicit recommendations in the discussion or conclusion for interventions tailored to this context. Most of the recommendations mentioned (PA/healthy eating in schools, built environment to promote PA) have been well studied and documented in other contexts and are not novel or innovative. In the revision, I suggest the authors consider what this data adds to the literature and how it can be used to inform intervention development.
We have revised our discussion and conclusion as suggested by the reviewer.

10) Various grammatical errors were noted throughout the manuscript (see comments in attached PDF). Suggest careful re-reading and editing for clarity.
We have revised the manuscript as suggested by the reviewer.

Reviewer #2:
Please see the review comments in the PDF document. I have highlighted the comments.

Main comments in the pdf
Introduction
1.Authors should take a paragraph or two to summarize the studies in Asia populations. This would help to demonstrate why a specifically cultural focus/regional focus is important.
We have added a paragraph focusing on Asian studies in the revised manuscript.

2.At some point, authors need to explain why the study reported here is focused on general health behaviors vs. Diabetes related health behaviors, as the parent study is a diabetes study. Otherwise, authors are encouraged to situate these findings within health behaviors around Diabetes.
As we have stated in the revised manuscript the intent of the study was to examine barriers and faciltators to healthy lifestyle in the broader context of general health behaviors which have been promoted at the policy level in Singapore. While the larger study was towards knowledge, attitudes and practices towards diabetes, the questions on lifestyle were general and not towards diabetes related health behaviors (Koh YS, Asharani PV, Devi F, Roystonn K, Wang P, Vaingankar JA, Abdin E, Sum CF, Lee ES, Müller-Riemenschneider F, Chong SA, Subramaniam M. A cross-sectional study on the perceived barriers to physical activity and their associations with domain-specific physical activity and sedentary behaviour. BMC Public Health. 2022 May 26;22(1):1051. doi: 10.1186/s12889-022-13431-2). This was also in consultation with diabetologists who opined that diabetes was often associated with other comorbid conditions and risk factors for several NCIDs were common. Thus, it was decided to understand general health behaviors. We have clarified this in the methodology.

Methodology

3.Please clarify: was this sampling method used for the parent study AND the interview component or both or just the interview component? If a specific sampling method was conducted (were there only 30 people who agreed to be recontacted from the survey? Probably not). So how did authors select FOR RECRUITMENT those who said they'd be willing to be contacted.
We have clarified the sampling method for the parent study. For the current study the participants were sampled randomly from those who gave consent for recontact. We have clarified the process in the methodology.

4.Are authors claiming that there were no a priori coding structures? If so, why use framework analysis?
We would like to respectfully state that a framework analysis can be used without apriori coding structures. We have further expanded the steps to explain what we did.

Powered by Editorial Manager® and ProduXion Manager® from Aries Systems Corporation

	While our initial coding was guided by our research questions and hence the topic guide, we did not use apriori coding.<br>Framework analysis in applied research have varied from highly deductive analysis (Pope et al., 2000) to inductively-oriented approaches in healthcare research (e.g., Goldsmith et al., 2017; Swallow et al., 2011).<br><br>Results<br><br>5.Specify cis, trans, nonbinary gender participants.<br>None of the participants identified themselves as cis/ trans or non-binary.<br><br>6.Authors should describe more about the participants in this sample: rurality, occupation (or employment), wealth or income, some measure of health (healthy weight? Health behavior such as smoking?). Readers need to understand more about the sample for this interview cohort.<br>We have described the sample in Table 1. Singapore is a highly urbanised city-state, there is no rural area in Singapore. We did not link health data to the qualitative interviews, and we have acknowledged it as a limitation of the study. We have added education and employment data.<br><br>Overall, the paper does not show the novelty of this study, even though (as argued but not substantiated) that Singapore has yet to be the focus of such a study.<br>We have revised our introduction to substantiate the novelty of the study.<br><br>If authors proceed with a revision, care and clarification is necessary with the manuscript revision to demonstrate study rigor and allow for replication. Further, authors should revisit the discussion and conclusion to 1) enhance the limitations section, and 2) truly demonstrate why this study is novel and important in PLOS One<br>We have made the changes suggested by the reviewers.<br><br>We hope that we have addressed the reviewers' comments adequately and we look forward to a favourable reply.<br><br>Regards<br>Authors
**Additional Information:**	
**Question**	**Response**
**Financial Disclosure**<br><br>Enter a financial disclosure statement that describes the sources of funding for the work included in this submission. Review the submission guidelines for detailed requirements. View published research articles from *PLOS ONE* for specific examples.<br><br>This statement is required for submission and **will appear in the published article** if the submission is accepted. Please make sure it is accurate.	SAC received the funding<br>NMRC/HSRG/0085/2018<br>This study is supported by the Singapore Ministry of Health's National Medical Research Council under its Health Services Research Grant<br>https://www.nmrc.gov.sg/who-we-are<br><br>The funders had no role in study design, data collection and analysis, decision to publish, or preparation of the manuscript.

Powered by Editorial Manager® and ProduXion Manager® from Aries Systems Corporation

**Unfunded studies**<br>Enter: *The author(s) received no specific funding for this work.*<br><br>**Funded studies**<br>Enter a statement with the following details:<br>• Initials of the authors who received each award<br>• Grant numbers awarded to each author<br>• The full name of each funder<br>• URL of each funder website<br>• Did the sponsors or funders play any role in the study design, data collection and analysis, decision to publish, or preparation of the manuscript?<br>• **NO** - Include this sentence at the end of your statement: *The funders had no role in study design, data collection and analysis, decision to publish, or preparation of the manuscript.*<br>• **YES** - Specify the role(s) played.<br><br>* typeset	
**Competing Interests**<br><br>Use the instructions below to enter a competing interest statement for this submission. On behalf of all authors, disclose any competing interests that could be perceived to bias this work—acknowledging all financial support and any other relevant financial or non-financial competing interests.<br><br>This statement is required for submission and **will appear in the published article** if the submission is accepted. Please make sure it is accurate and that any funding sources listed in your Funding Information later in the submission form are also declared in your Financial Disclosure statement.<br><br>View published research articles from *PLOS ONE* for specific examples.	The authors have declared that no competing interests exist.

**NO authors have competing interests**<br><br>Enter: *The authors have declared that no competing interests exist.*<br><br>**Authors with competing interests**<br><br>Enter competing interest details beginning with this statement:<br><br>*I have read the journal's policy and the authors of this manuscript have the following competing interests: [insert competing interests here]*<br><br>* typeset	
**Ethics Statement**<br><br>Enter an ethics statement for this submission. This statement is required if the study involved:<br><br>• Human participants<br>• Human specimens or tissue<br>• Vertebrate animals or cephalopods<br>• Vertebrate embryos or tissues<br>• Field research<br><br>Write "N/A" if the submission does not require an ethics statement.<br><br>General guidance is provided below. Consult the submission guidelines for detailed instructions. **Make sure that all information entered here is included in the Methods section of the manuscript.**	Human Subject Research<br>National Healthcare Group Institutional Review Board DSRB Ref: 2019/00926<br>All subjects provided written informed consent

Powered by Editorial Manager® and ProduXion Manager® from Aries Systems Corporation

**Format for specific study types**<br><br>**Human Subject Research (involving human participants and/or tissue)**<br>• Give the name of the institutional review board or ethics committee that approved the study<br>• Include the approval number and/or a statement indicating approval of this research<br>• Indicate the form of consent obtained (written/oral) or the reason that consent was not obtained (e.g. the data were analyzed anonymously)<br><br>**Animal Research (involving vertebrate animals, embryos or tissues)**<br>• Provide the name of the Institutional Animal Care and Use Committee (IACUC) or other relevant ethics board that reviewed the study protocol, and indicate whether they approved this research or granted a formal waiver of ethical approval<br>• Include an approval number if one was obtained<br>• If the study involved *non-human primates*, add *additional details* about animal welfare and steps taken to ameliorate suffering<br>• If anesthesia, euthanasia, or any kind of animal sacrifice is part of the study, include briefly which substances and/or methods were applied<br><br>**Field Research**<br><br>Include the following details if this study involves the collection of plant, animal, or other materials from a natural setting:<br>• Field permit number<br>• Name of the institution or relevant body that granted permission	
**Data Availability**<br><br>Authors are required to make all data underlying the findings described fully available, without restriction, and from the time of publication. PLOS allows rare exceptions to address legal and ethical concerns. See the PLOS Data Policy and FAQ for detailed information.	No - some restrictions will apply

Powered by Editorial Manager® and ProduXion Manager® from Aries Systems Corporation

A Data Availability Statement describing where the data can be found is required at submission. Your answers to this question constitute the Data Availability Statement and **will be published in the article**, if accepted.<br><br>**Important:** Stating 'data available on request from the author' is not sufficient. If your data are only available upon request, select 'No' for the first question and explain your exceptional situation in the text box.<br><br>Do the authors confirm that all data underlying the findings described in their manuscript are fully available without restriction?	
**Describe where the data may be found in full sentences. If you are copying our sample text, replace any instances of XXX with the appropriate details.**<br><br>• If the data are **held or will be held in a public repository**, include URLs, accession numbers or DOIs. If this information will only be available after acceptance, indicate this by ticking the box below. For example: *All XXX files are available from the XXX database (accession number(s) XXX, XXX.)*.<br>• If the data are all contained **within the manuscript and/or Supporting Information files**, enter the following: *All relevant data are within the manuscript and its Supporting Information files.*<br>• If neither of these applies but you are able to provide **details of access elsewhere**, with or without limitations, please do so. For example:<br><br>*Data cannot be shared publicly because of [XXX]. Data are available from the XXX Institutional Data Access / Ethics Committee (contact via XXX) for researchers who meet the criteria for access to confidential data.*<br><br>*The data underlying the results presented in the study are available from (include the name of the third party*	Data cannot be shared publicly because of ethical and institutional regulations. Data are available from the Institute of Mental Health Institutional Research Review Committee (contact via research@imh.com.sg) for researchers who meet the criteria for access to confidential data.

Powered by Editorial Manager® and ProduXion Manager® from Aries Systems Corporation

and contact information or URL).<br>• This text is appropriate if the data are owned by a third party and authors do not have permission to share the data.<br><br>* typeset	
Additional data availability information:	Tick here if your circumstances are not covered by the questions above and you need the journal’s help to make your data available.

Powered by Editorial Manager® and ProduXion Manager® from Aries Systems Corporation

5 May 2022

Dr. Emily Chenette
Editor- in- Chief
PLOS ONE

Ref: Barriers and Facilitators for adopting a healthy lifestyle in a multi-ethnic population:

A qualitative study

Dear Dr Chenette,

We are submitting the above-mentioned paper for your consideration for publication as an original research article. This article examines the barriers and facilitators for adopting a healthy lifestyle in a multi-ethnic population. The article is especially important as Singapore has rolled out a nation-wide War on Diabetes campaign which aims to improve the lifestyle of the population to reduce diabetes burden. The results of our study can lead to the strengthening of the efforts of this campaign. The strengths of our study include that we conducted interviews in English and local languages and used the socio-ecological model as a framework to understand the factors.

This manuscript has been read and approved by all the authors, and it has not been published elsewhere. The study was approved by the relevant ethics Committee (National Healthcare Group, Domain Specific Review Board) and all participants gave written informed consent. The study has not been published or under consideration elsewhere.

We look forward to a favourable response from you.

Sincerely

Mythily Subramaniam
Research Division, Institute of Mental Health
Buangkok Green Medical Park, 10 Buangkok View
Singapore 539747
E-Mail: Mythily@imh.com.sg
Telephone number: 6563893633

Manuscript Click here to access/download;Manuscript;Manuscript .docx

Title: Barriers and Facilitators for adopting a healthy lifestyle in a multi-ethnic population: A qualitative study

Authors: Mythily Subramaniam[1,2], Fiona Devi[1], P.V. AshaRani [1], Yunjue Zhang[1], Peizhi[1], Anitha Jeyagurunathan[1], Kumarasan Roystonn[1], Janhavi Ajit Vaingankar[1], Siow Ann Chong[1]

[1]Research Division, Institute of Mental Health, Singapore

[2]Saw Swee Hock School of Public Health, National University of Singapore

Corresponding Author: Mythily Subramaniam

Assistant Chairman Medical Board (Research)

Research Division, Institute of Mental Health, Buangkok Green Medical Park, 10 Buangkok View, Singapore 539747,

Tel: 65-63893633 Fax: 65-6343 7962, Mythily@imh.com.sg

Abstract

A healthy lifestyle is defined as 'a way of living that lowers the risk of being seriously ill or dying early.' Although barriers and facilitators of healthy lifestyles have been well-studied among populations like those with chronic non-communicable diseases, adolescents, and older adults in Asia, less information is available on barriers and facilitators perceived by the general adult population. The current study leveraged the socio-ecological model to gain a deeper understanding of the barriers and facilitators of a healthy lifestyle in a sample of Singapore residents using a qualitative methodology. In all 30 semi-structured interviews were conducted in English and other local languages from August 2020 to March 2021. Transcripts were analysed using framework analysis. Five main themes pertaining to personal, interpersonal, environmental, socio-cultural, and policy-level factors were classified under the two overarching categories of barriers and facilitators of healthy lifestyles. The results of this study offer important insights into understanding the barriers and facilitators to the adoption of a healthy lifestyle among people in Singapore. Our findings illustrate the complex interplay between individuals, social relationship, environment, and policy that can act as either a barrier or a facilitator to adopting a healthy lifestyle.

Key words: healthy lifestyle; willpower; family; technology; qualitative

Introduction

Life expectancy at birth has increased globally, and improvements in population health status have been observed for every life stage [1]. Public health initiatives such as universal immunisation, enhanced health infrastructure, improved maternal and infant health, tobacco control, and motor vehicle safety have driven these changes globally [2]. This increased life expectancy is associated with changing disease patterns, i.e., a transition from acute to chronic diseases as the primary source of morbidity and mortality worldwide [3]. At the same time, the physician-patient relationship has progressively moved towards shared decision-making, where clinicians and patients make decisions together using the best available evidence [4]. Furthermore, with patients becoming avid consumers of medical knowledge and taking on a more active role in their wellbeing, the responsibility for health rests on individuals and societies [5].

The World Health Organisation (WHO) defines a healthy lifestyle as 'a way of living that lowers the risk of being seriously ill or dying early' [6]. Pender's health promotion model defines barriers to a healthy lifestyle as 'factors that directly interfere with the enactment of a health-promoting behavior or mediate by reducing the commitment to the plan of action for changing behavior' [7]. On the other hand, facilitators are defined as factors that promote or enable the uptake and maintenance of a healthy lifestyle [8]. A systematic review of 32 studies from 1980 to February 2010 by Murray et al. [9] found that a better understanding of illness, and perception of significant consequences of illness, were strong facilitators that promoted the uptake of lifestyle behavior change interventions. While stress, depression, lack of social support, and transport and commute time problems were significant barriers. Similar findings were reported by Kelly et al. [10] in their rapid systematic review, which examined barriers and facilitators to the uptake and maintenance of healthy behaviours by people in their mid-life. Across 28 qualitative studies, 11 longitudinal cohort studies, and 46 systematic reviews,

the authors identified several barriers. These included lack of time, access (to transport, facilities, and resources), financial costs, entrenched attitudes and behaviours, low socioeconomic status, and lack of knowledge. In contrast, facilitators included enjoyment and a sense of wellbeing associated with physical activity, health benefits including healthy ageing, social support, clear messages, accessible websites, and previous experience of ill-health.

Although barriers and facilitators of healthy lifestyles have been well-studied among populations like those with chronic non-communicable diseases, adolescents, and older adults in Asia, less information is available on barriers and facilitators perceived by the general adult population. A qualitative study from Sri Lanka identified several barriers to adopting physical activity in an urban activity-friendly area. The barriers included competing priorities like work, physical concerns like safety, health concerns such as discomfort, resources including facilities and social support, and lack of understanding of the importance of physical activity for health [11]. A study from Nepal that examined the barriers and facilitators to healthy eating in a worksite cafeteria identified unavailability or high cost of healthy foods, cultural preferences for fried food, and difficulty in changing eating habits as the main barriers. This study identified the availability and affordability of healthy food as the main facilitators of healthy eating [12]. A qualitative study conducted in India among the attendees of an urban health centre identified motivation/willpower, time management skills, knowledge and perceived benefits of physical activity, health problems, and availability of exercise facilities as facilitators of physical activity. The lack of time, space, or equipment, unfavourable weather, physical restriction, and laziness were important barriers [13]

Singapore is a densely populated, urbanised, city-state in Southeast Asia with a multiracial population of about 5.5 million, comprising Chinese, Malays, Indians, and a smaller proportion of other ethnicities [14]. The country has a highly developed free-market economy and a gross domestic product per capita of about US$60,000 in 2020 [15]. Between 1990 and 2017, life

expectancy in Singapore rose from about 76.1 years to 84.8 years; however, healthy life expectancy at birth increased only by 7.2 years to 74.2 years (16).

The Singapore Government has focused on promoting and providing health beyond healthcare facilities. It has made concerted efforts and worked consistently across sectors to promote evidence-based practices to build and maintain a culture of active living in the population [17]. Drawing upon the 7 Investments that Work (7IW) framework [18], the government has worked on transport policies, public education, healthcare, and urban design initiatives to promote physical activity. In addition, Singapore has encouraged the philosophy of individual responsibility, i.e., personal responsibility remains a central tenet of Singapore's approach to healthcare (16).

Furthermore, in 2016, the Singapore Health Minister declared War on Diabetes (WoD). The effort was mainly in response to the higher prevalence of diabetes in Singapore compared to the global prevalence rate, with nearly one in ten Singaporeans (9.5%) suffering from the disease [19]. The WoD had three main thrusts, with the first thrust being prevention through healthy living. As part of this thrust, the government increased the availability and accessibility of physical activity programmes, launched nationwide physical activity-based challenges, and increased the availability of healthier food options in schools, restaurants, and food courts [20]. Thus, against this backdrop of sustained health-promoting policies and the focus on personal responsibility for health, Singapore presents a unique opportunity to understand the barriers and facilitators for the uptake of a healthy lifestyle.

Several theoretical frameworks have been offered to explain health behavior. The socio-ecological model has been widely used to understand the interrelations between personal, social, and environmental determinants of lifestyle behavior [21,22]. The model is attractive as it incorporates a wide range of influences at multiple levels, and variables included in ecological models comprise intrapersonal or personal (biological, psychological), interpersonal/cultural,

organizational, physical environment (built, natural), and policy (laws, regulations). We adopted the perspective of the SEM framework to gain a deeper understanding of the barriers and facilitators of a healthy lifestyle in Singapore, given that personal, cultural (multi-ethnic population), and environmental (backdrop of nationwide campaign and initiatives) factors would substantially influence the population's lifestyle. A secondary aim of the study was to explore if the people were aware of and utilized the interventions launched as part of the WoD to improve healthy lifestyles. The findings of such a study can help develop targeted interventions to overcome the barriers and enhance the facilitators to improve the impact of national campaigns in Singapore.

Methods

Study Design and Setting

The data for the current study was part of a more extensive study that examined the knowledge, attitudes, and protective practices toward diabetes among the public in Singapore. The study comprised a quantitative survey (n=2895) and a qualitative phase (n=30) to explore the barriers and facilitators of a healthy lifestyle in Singapore. The study methodology was published in an earlier article [23]. The sample for the nationwide survey was derived using a disproportionate stratified sampling design. In all, 12 strata: a combination of 3 strata for ethnicities (Chinese, Malay, and Indian) and 4 strata for age (18 to 34 years, 35 to 49 years, 50 to 64 years, and 65 years and above) were employed for the sampling. The proportion of respondents in each ethnic group (Chinese, Malay, and Indian) was set at approximately 30%, while the proportion of respondents in each age group was set at around 20% to ensure a sufficient sample size for these population subgroups [23].

The participants for the qualitative study were recruited from among those who participated in the quantitative survey and permitted recontact for future research studies. The inclusion

criteria comprised Singapore citizens or permanent residents aged 21 years or above, able to speak in either English, Chinese, Malay, or Tamil, and not diagnosed by a doctor as having diabetes. Participants were stratified according to their age ($\geq$ 40 years and < 40 years), gender, and ethnicity (Chinese, Malay, Indians, and others) and then randomly chosen using an online randomisation software and recruited into the qualitative phase. To account for participant refusals, the sample was drawn in excess (i.e., 60 English-speaking participants and 30 native language speakers). This sampling allows multiple perspectives to be presented that illustrate the complexity of the phenomenon under study [24]. The researchers did not link the quantitative data provided by the individuals with the qualitative data.

Data Collection

In Singapore, The Government imposed a nationwide ‘circuit breaker’ comprising restrictions on public gatherings and dining in restaurants, home-based schooling and working from 7th April 2020 until 1st June 2020. As of 2nd June 2020, Singapore entered the ‘reopening’ phase, and businesses and activities were progressively allowed to operate. The study period for the current study was eight months, from August 2020 to March 2021. While the measures had been relaxed, and a significant proportion of the population was vaccinated, some participants were uncomfortable doing face-to-face interviews. Interviews were therefore conducted in person or via the Zoom video-conferencing platform, depending on the participants’ preference. In all, 30 interviews were conducted; 20 were in English, four in Chinese, and three in Malay and Tamil. Each interview lasted between 60-90 minutes and was audio-recorded. Participants were interviewed in venues that afforded privacy so they could freely express their views. Participants who opted for a Zoom interview were similarly informed that they should ensure a quiet and private interview setting. Each interview was conducted by a trained qualitative interviewer, accompanied by a note-taker, using an interview guide.

The interview guide was developed based on existing literature [25, 26] and further modified after discussions with a general practitioner and a diabetologist. The researchers minimally modified the interview guide to include relevant probes after the first two interviews (Supplementary File 1). A guided discussion format was used, and participants were encouraged to speak freely about their thoughts and experiences [27].

Data analysis was undertaken concurrently, allowing emerging themes to inform ongoing data collection. The researchers met up regularly and discussed emerging findings to ensure the trustworthiness of the data. Data collection ceased when saturation could reasonably be assumed. The team members transcribed and analysed the data after recruiting the first 20 English-language interviews before commencing the local-language interviews. This was to ensure that we had reached thematic saturation with the data collection and to simultaneously assess the language-specific interviews for the emergence of any new themes. An additional ten interviews were conducted with native speakers, i.e., those able to converse only in Chinese, Malay, and Tamil. This ensured that the perspectives of those belonging to a different socio-cultural background were taken into consideration.

Written informed consent was taken from all the participants, and ethical approval for the study was obtained from the relevant institutional review board (National Healthcare Group Domain Specific Review Board; protocol ref:2019/00926).

Qualitative analysis

The interviews (English and language-specific) were transcribed or translated and transcribed verbatim by a professional transcription firm and checked for accuracy by a study team member. Transcripts were analysed using framework analysis. The framework method was originally developed for use in large-scale policy research [28], however it is now used widely in healthcare research. It is a data analysis method rather than a research paradigm

which, unlike entirely inductive and iterative approaches, may be shaped by existing ideas and is less focused on producing a new theory [29].

Data Familiarisation

First, seven researchers (ZYJ, AR, FD, WP, KR, AJ, and MS) familiarised themselves with the first eight transcripts by reading them multiple times. This was in line with Srivastava and Thomson (30), who stated that, given the large volume of data in qualitative research, not every piece of material may be reviewed at this stage. Following the deep reading, initial themes were identified by individual researchers. These themes were checked against the interview guide and study objectives, resulting in the development of a set of preliminary codes for different barriers and facilitators to a healthy lifestyle.

Constructing an initial thematic framework

The researchers then met to discuss and combine their preliminary codes. These discussions helped in resolving disagreements in defining or including themes. Largely there was consensus among the team members, but when there was a disagreement that could not be resolved, the first author made a call on the inclusion and definition of initial themes. These initial codes were then sorted into a hierarchy of themes and sub-themes to construct an initial framework. To ensure that all the research objectives were met, the initial framework consisted of four main categories, with several sub-themes under each category.

On reaching a consensus, a codebook was constructed, which contained a detailed description of each code, the inclusion and exclusion criteria, and typical and atypical exemplars to assist with valid and reliable code application.

Indexing and sorting

Each semi-structured interview (SSI) was used as a unit of analysis. To determine 'what parts of the data are about the same thing and belong together' (31), labels were applied to 'chunks' of data with the same meaning to decide the category/ theme from the framework to assign the

text to. Using NVivo, the selected (highlighted) text was ‘dragged and dropped’ into the relevant sub-themes. This process was followed for all the transcripts.

Three interviewers (AR, FD, and WP) systematically applied the framework to all the transcripts after achieving an inter-rater reliability of 0.87 (Kappa (κ) value) with the first two transcripts. There were no significant disagreements between the three coders on any subthemes or categories.

Mapping and Interpretation

The finalized themes and subthemes were grouped together. Once the main themes and sub-themes were reviewed and finalised, a matrix was created for each theme using Excel, with individual columns for the sub-themes. The first column of the matrix contained case identification details (demographics), followed by summaries of individual themes in subsequent columns. Representative quotes were selected from the SSI to illustrate key themes and subthemes. These themes and subthemes are represented pictorially in Figure 1.

All analyses were conducted using Nvivo V.11 (QSR International. NVivo V.11).

Results

A total of 30 participants (14 females and 16 males) participated in the study. The mean age of participants was 44.7 years (SD=14.7), ranging from 21 to 75 years (Table 1).

Table 1: Socio-demographic profile of participants

Subject ID	Age	Ethnicity	Gender	Language of interview	Education	Employment
SS001	25	Chinese	F	English	Diploma	Employed
SS002	24	Chinese	M	English	Diploma	Student
SS003	39	Malay	F	English	Diploma	Employed
SS004	38	Malay	F	English	Post graduate degree	Homemaker
SS005	54	Chinese	F	English	Degree	Employed
SS006	25	Malay	M	English	Diploma	Employed
SS007	34	Malay	M	English	Completed secondary education	Employed
SS008	27	Malay	M	English	Degree	Employed
SS009	31	Malay	F	English	Degree	Employed
SS010	22	Chinese	M	English	Completed high school (equivalent)	Student
SS011	53	Chinese	F	English	Completed high school (equivalent)	Employed
SS012	55	Malay	F	English	Completed primary education	Employed
SS013	56	Chinese	M	English	Diploma	Unemployed
SS014	26	Chinese	F	English	Degree	Employed
SS015	46	Others	F	English	Post graduate degree	Employed
SS016	38	Chinese	M	English	Vocational Institute Training	Unemployed
SS017	24	Others	M	English	University degree	Employed
SS018	58	Indian	M	English	Completed secondary education	Employed
SS019	52	Indian	M	English	Post graduate degree	Employed
SS020	54	Indian	M	English	Degree	Employed
SS021	60	Chinese	M	Chinese	Some secondary education	Employed
SS022	39	Chinese	M	Chinese	Degree	Employed
SS023	60	Chinese	M	Chinese	Completed secondary education	Unemployed
SS024	59	Chinese	F	Chinese	Completed secondary education	Employed
SS025	54	Malay	F	Malay	Completed primary education	Unemployed
SS026	71	Malay	M	Malay	Completed primary education	Homemaker
SS027	65	Malay	F	Malay	Some secondary education	Homemaker
SS028	37	Indian	M	Tamil	Completed secondary education	Employed
SS029	61	Indian	F	Tamil	Completed primary education	Employed
SS030	53	Indian	F	Tamil	Some secondary education	Homemaker

Five main themes pertaining to personal, interpersonal, environmental, socio-cultural, and policy-level factors were classified under the two overarching categories of barriers and facilitators of healthy lifestyles. The personal, interpersonal, environmental, and policy-level factors comprised subthemes and are highlighted below. Figure 1 shows the summary of the findings of the themes and sub-themes. Minimally edited verbatims have been included that

preserve and highlight the participants' experiences and beliefs. The details of the participants have been provided in brackets as Subject ID/Age/ Gender (**M**ale or **F**emale).

<u>Barriers to a Healthy Lifestyle</u>

Personal Factors

Personal factors comprised two main subthemes explaining the barriers to adopting a healthy lifestyle. These included:

Lack of willpower and self-discipline

About one-third of participants mentioned the lack of willpower as an important barrier to maintaining a healthy lifestyle in terms of diet and exercise. They described people as 'being lazy,' using 'tired,' 'too busy,' and 'work' as an excuse not to partake in physical activity. They also alluded to personal dietary preferences such as liking sweets and desserts and people not having the willpower to resist them. A few of the respondents felt that people were aware of the ill effects of consuming too much sugar, yet they did not have the self-discipline to limit their intake. In addition, respondents felt that people generally knew about the negative outcomes of smoking and alcohol. Yet, they did not quit smoking/ drinking as they did not have the willpower to do it and instead made-up excuses when asked to quit. Respondents also felt that while people may have good intentions and want to adopt a healthy lifestyle, they lacked the willpower to follow through and fell back into unhealthy habits.

"It is your own self-cultivation and self-discipline that has to do with your health. If you don't have good self-discipline and you mess around, what kind of healthy body will you have?" (SS022/39/M)

"It's really hard to change. I have been in contact with a few of them (referring to smokers). One of them stopped smoking for three months. "I stopped smoking." After a few days, he

started again. I asked him, 'What do you smoke for?' "Oh, pressure." Pressure is fake." (SS021/60/M)

Lack of knowledge

Participants felt that it was difficult for someone with a chronic health condition to exercise. They also felt that as a person becomes older, they should avoid vigorous exercise as they could injure themselves more easily. Thus, they thought it was advisable for older people and those with chronic health conditions to reduce their exercise. They did not seem aware that both these groups could exercise safely and substitute high-impact activities for lower-impact ones. Regarding diet, respondents expressed frustration with contradictory messages on what was healthy. They felt that food once considered healthy was no longer believed to be healthy and vice versa. Thus, they were unsure what should be consumed and what should be avoided. A few respondents identified social media as a significant source of unreliable health information.

"For those who have health conditions, it is very difficult for them to do exercise daily. They are in a life situation where they just cannot take part in a lot of things." (SS030/53/F)

"… especially those messages on social media and WhatsApp about your health. Some people say, "Don't take coconut." Some people say, "Yeah. Coconut is healthy. You take more coconut." So really difficult to judge which is right or which is wrong." (SS019/52/M)

Interpersonal Factors

Negative attitudes and negative influences by family and friends towards a healthy lifestyle

Interpersonal factors were mainly identified as the negative attitudes of family and friends towards a healthy lifestyle or the influence of unhealthy practices of friends and family members. E.g., participants mentioned over-eating during family occasions as there was an

excess of food and family members or friends urged them to eat more during social gatherings. Participants said that friends who ridiculed their healthy eating habits were barriers to adopting a healthy lifestyle. Several participants shared that when they have food with friends, they tend to over-eat or eat food that is not particularly healthy. They felt uncomfortable not eating the food as they were afraid to be perceived as spoilsports if they did not partake in the feasting and drinking.

"I think there's a stigma against healthy food. I know how some of my friends say that eating your salad is girly or whatnot. So maybe eating salad is associated with teenage girls, I guess. I don't know. And I guess maybe if they smoke a stick or drink beer, it is more associated with masculine ideals. So maybe if you don't follow the party or whatnot, you may be viewed as an outlier or something. So yeah. I would say there is some social stigma tied to certain types of food or lifestyle, I guess." (SS010/22/M)

"...for example, go out with friends and drink milk tea (referring to bubble milk tea, a sweet tea with tapioca balls that is very popular in Singapore) together, and then he drinks a cup and buys you a cup. Will you not drink it? Sometimes, it's not very polite to say no to your friends." (SS022/39/M)

Environmental factors

As most of the interviews were conducted during the COVID-19 pandemic when outdoor activity was restricted, it was not surprising that participants mentioned the pandemic as a significant barrier to physical activity. The fear of infection, safe distancing measures, and other restrictions hindered participants from performing outdoor activities and those conducted in gyms or enclosed spaces. The other sub-theme that emerged pertained to situational factors such as conflicting demands leading to time constraints and the low cost and ready availability of fast food that were perceived to be barriers to adopting a healthy

lifestyle.

COVID-19 pandemic

All the participants mentioned the impact of the COVID-19 pandemic on their lifestyles and expressed their fears and frustrations. For example, participants talked about how the social distancing regulations, closure of indoor gyms and training spaces, and the need to mask up (even in outdoor spaces in Singapore) were significant barriers to exercising in Singapore. Moreover, putting a mask on for most of the day in Singapore's hot and humid weather left them feeling tired, irritable, and reluctant to exercise.

"Actually, before COVID, my friends and I played soccer on a weekly basis, every Sunday. Yeah. But because of COVID, then we stopped completely." (SS008/27/M)

"And looking at the number of cases (Covid cases), they went up higher. So, it's a deterrence to exercising." (SS022/39/M)

Situational factors

The most discussed barrier was time constraints associated with competing priorities such as employment, household chores, and looking after children or older parents. However, other factors, such as financial constraints and limited access to healthy food, were also reported to impact healthy lifestyle behaviours.

Participants mentioned that healthy food was both expensive and not readily available. They acknowledged that fast food was the most convenient food, and while they knew that consuming a diet rich in calories was associated with being overweight, they were unable to avoid it. The easy availability of fast food at all hours and food stalls that stayed open even during the night in Singapore also encouraged poor food habits. Interestingly, food delivery was associated with an unhealthy lifestyle. Participants felt that food delivery led people to

order more due to convenience and easy availability. In addition to that, it also led to them eating at odd hours.

While most participants acknowledged that Singapore had several parks and exercise areas that were conducive to physical activity, three of the respondents had concerns about the safety of these facilities. These included dimly lit parks that made walking difficult at night, sharing of the same path by pedestrians, cyclists, and children and teenagers who tended to run or skate, thus increasing the risk of accidents among older adults. In addition, two participants highlighted the lack of good cycling tracks in Singapore, which does not encourage a cycling culture, unlike Denmark or the Netherlands.

"Because sometimes, frankly speaking, children are young, and it is hard for you to have any free time for yourself." (SS024/59/F)

"I think maybe the fact that healthy food is quite expensive in Singapore. So, I guess food, in general, can be quite affordable if you go hawker centre (hawker centres are open-air complexes with many stalls that sell a wide variety of affordably priced food) *or whatnot. But then they are generally not very healthy, so it can be quite troublesome for some people to cook healthier food." (SS010/22/M)*

"Now you can even use your phone to just order, and they will deliver it directly to your house. Ordering food or eating outside food has become so easy that it has become a part of their lifestyle. So, they don't give much consideration to the food itself, and a healthy lifestyle is lost." (SS030/53/F)

Cultural factors

Several cultural factors emerged as barriers to the adoption of healthy lifestyles. Given the multi-ethnic nature of Singapore, barriers pertaining to cultural factors were identified both by people belonging to that ethnocultural group and others. These included:

The cultural importance of traditional food

Participants acknowledged that cooking and eating traditional food was an important ritual in Singapore. However, about one-third of the participants felt that Indian and Malay food tended to be oily and calorie-rich. They also acknowledged that these types of food appealed to people's tastes, and they ended up overeating them. In addition, they felt that desserts unique to these cultures were similarly sweet and not healthy. Coconut milk in traditional food preparations was similarly identified as an unhealthy but necessary ingredient. Some also commented that those of the Chinese ethnicity liked to eat pork and were unwilling to switch to healthier meat alternatives (such as white meat). They also acknowledged that Chinese cuisine could be oily as many dishes are deep-fried.

"In fact, Singapore's food is not only western food, but also Malay food, Indian food, and Chinese food. It doesn't contain as much oil as Chinese cuisine in China. Chinese food has a lot of oil. The Chinese food here, oil and salt, will not be so overused, but it will have a lot of fried things. And your Indian food and Malay food, I believe it, will have a lot of sugar, especially Malay food." (SS022/39/M)

Customs and Festivals

Participants also talked about food habits of specific ethnic groups, such as eating dinner late at night and close to bedtime, which they perceived as unhealthy. They also acknowledged that festive periods were not conducive to maintaining a healthy lifestyle as it was all about meeting friends and families and eating. So, one tended to over-eat during such periods.

"As far as we Chinese are concerned, if we talk about the Chinese New year, it may be that everyone eats more…" (SS021/60/M)

Language barriers

People from minority ethnicities expressed their reluctance to participate in community-based group exercise programs as they felt they would not be able to understand the instructions as these tend to be conducted in a language, they are not conversant.

"…that's why I just hate to go to some of these community activities. They are all in English, and I won't understand. Most or all are in English only, so I feel a little uncomfortable because of that." (SS030/50/F)

Policy related factors

Policy-related barriers did not emerge very strongly in this group of participants. However, a few participants expressed their frustration with the reluctance of the government to impose a sugar tax. They felt that sugar caused significant harm to a person's health, but it was not something that could be taxed. They mentioned the 'bubble tea fad' in Singapore, which led to several shops across the country selling sweet and calorie-rich drinks. The existing policies could not limit such shops; the government they felt could only advocate and educate people about the potential harms of such food.

"I don't think it's realistic. Because, for example, the government tells people not to smoke, then they increase the tax on cigarettes. Don't drink, and they will add a high tax to wine. But it is impossible to add a high tax on sugar because sugar is a necessity in life. Unlike tobacco and wine, it is not a luxury but a part of the diet. Many people in their daily life use sugar. So, you can't, the government can't say, add a high tax to milk tea shops. So, I think from the government's point of view that they can't do many things. They can just advocate." (SS022/39/M)

<u>Facilitators of a Healthy Lifestyle</u>

Personal and Interpersonal factors

Most of the facilitators highlighted by the participants in these two themes were the opposite

of those mentioned as barriers. However, the absence of a barrier was not necessarily a facilitator. For example, most participants highlighted ‘willpower and motivation’ as personal facilitators. Participants talked about how willpower was necessary to exercise regularly and eat healthy food. They also felt that if people knew the impact of a healthy lifestyle on long-term outcomes, they would commit to them. One-third of participants also acknowledged that people with health conditions should continue to exercise and maintain a healthy diet as it can prevent secondary complications. They also highlighted the important role of friends and family members in encouraging and supporting a healthy lifestyle, which helped the participants maintain it.

Organisational/ Institutional factors

Workplace-initiated health promotion interventions

The workplace emerged as a significant facilitator of a healthy lifestyle. More than half of the participants who were employed mentioned various workplace initiatives that had helped them to become more physically active. This was mainly through workplace wellbeing initiatives such as the distribution of fruits, subsidized fruit bazaars at the place of work, educational sessions on diet and its impact on wellbeing, and group exercise classes like Zumba or Yoga. Many workplaces continued these initiatives even during the pandemic by leveraging on Zoom and other platforms.

"But my workplace, I would say they are trying to endorse the whole healthy lifestyle thing. So, we do have things like a monthly fruit giveaway. So, every single month we'll get different kinds of fruit and then staff will explain to us the benefit of eating food, or fruit rather. We have staff exercise sessions where you can sign up for yoga or gym sessions or go for a walk." (SS009/31/F)

Influence of healthcare and other professionals

Several participants talked about adopting a healthier lifestyle after their healthcare provider (usually a doctor or dietician) advised them about healthy eating or physical activity. They also spoke of informative media programs that encouraged the adoption of a healthy lifestyle.

"Polyclinics (primary care clinics) or I think if I didn't recall wrongly-- I can't really remember. Is it one of the hospitals or polyclinics my parents visited? They actually have a nutritionist who tells you what to eat." (SS016/38M)

"White meat, the doctor's advice is to eat more white meat instead of red meat because red meat is not good for cholesterol." (SS022/39/M)

Environmental factors

Role of Technology as a facilitator of a healthy lifestyle

All the participants highlighted the role of technology as a facilitator of a healthy lifestyle. However, their understanding of technology varied. Any source of information like the television, radio, Internet search engines, channels like YouTube, social media sites, and Apps (mobile applications) was described as technology. Participants saw technology as enabling access to information on diet and exercise, aiding in training and tracking and monitoring their physical activity, heart rate, sleep, and food consumption. Some participants alluded to Apps that sent reminders to breathe deeply, meditate, and walk as helpful.

"App for cycling that's called Strava. I think that's just the only healthy fitness app that I have. So, they will keep track of your heartbeat, the distance from one point to another, and the speed of cycling." (SS007/34/M)

"It's MyFitnessPal. Well, it tracks my calorie intake for the day. And it's quite specific. It's quite good. But I think they cannot detect some of the local foods. But other than that, they

can track basically whatever that goes in your mouth, yeah, whatever you consume. And you can put in your exercises for the day. So, yes, it will help you calculate your goals, like how many KG you want to lose in a month also, which is quite good." (SS008/24/M)

Policy-related factors

The participants highlighted several policy-related facilitators. These included:

Built spaces and exercise stations

More than half of the participants talked about the availability of neighbourhood parks which provided a safe and convenient place to exercise. Participants mentioned that walks were often organised in their neighbourhoods and that volunteers would encourage them to join in these activities. They were also aware that several activities were conducted in these spaces that one could join at no cost. Some also felt that such group activities motivated them, and they enjoyed doing these more than doing exercises by themselves. Participants also mentioned that there are public swimming pools that one could use and government-run gyms where one could access high-quality equipment at a minimal cost. Participants acknowledged that the government was constantly upgrading parks and gyms, and they could now easily access parks and exercise corners.

"Walking, our government is good, gave us many parks and so many connectors (scenic roads connecting parks where pedestrians can walk). You cannot say that there are no facilities." (SS024/59/F)

"Sometimes they do come and call me to join, like what's that called, social service Community volunteers. When we exercise with other people in a group, it gives us a sort of motivation." (SS030/53/F)

Inclusion of physical education as a core curriculum in school

About a quarter of the participants mentioned that including physical education in schools and

getting children to exercise regularly as part of the school curriculum encouraged incorporating exercise into their lifestyle. They also shared that even tertiary education institutes offered an excellent array of exercise classes/ options, which enabled the students to continue exercising. *"The education system right now really talks about mental health and physical health. Even their physical education is different. So, I think exposure to that will be one of the factors that will enable them to live a healthy lifestyle." (SS009/31/F)*

"So, when I went to XXX Poly [polytechnic name], I started Muay Thai. And even the gym was affiliated with XXX [polytechnic name]. So, I've been with this gym since the dawn of time. This is my first gym, and I've been with them all the way [laughter]." (SS006/25/F)

Figure 1 Barriers and Facilitators of adopting a healthy lifestyle

Discussion

This article explored the barriers and facilitators of a healthy lifestyle perceived and experienced by a multi-ethnic sample of adults in Singapore. Using a framework analysis approach that comprised two major components: creating an analytic framework and applying this analytic framework, we leveraged the SEM [22] model to gain a deeper understanding of the barriers and facilitators of a healthy lifestyle. The discussion focuses on key themes that lend well to intervention or were unique to this study.

At the personal level, lack of willpower emerged as a key barrier, while being motivated and having the resolve to exercise or not eat sweet or calorie-rich food, despite the challenges, was identified as a facilitator. Willpower, defined as the capacity to exert self-control, has emerged in several studies as a barrier to healthy eating [32,33] and physical activity [34]. In Tsukayama et al.'s [35] prospective longitudinal study, the researchers found that more self-controlled children were less likely to become overweight as they entered adolescence. Cognitive and behavioural interventions have been developed to promote self-regulation [36] and overcome

this barrier. For example, a study among women aged 30-50 showed that a brief intervention combining information with a self-regulation technique led to the maintenance of high consumption of fruits and vegetables 24 months after the intervention. In contrast, the information-only intervention group returned to baseline consumption of fruits and vegetables [37].

Similarly, another study tested an intervention that combined information with cognitive-behavioural strategies on women's physical activity with an information-only intervention. The women who were randomly assigned to the self-regulation and information session were substantially more active than those who participated in the information-only sessions [38]. While the WoD campaign has ensured the dissemination of information on a healthy lifestyle, there is a need to develop and evaluate interventions that provide information and teach and promote self-regulation. If such interventions are effective, they could be scaled up at the population level.

Environmental factors that emerged as barriers and facilitators were unique to this study. The study period coincided with the COVID-19 pandemic, and all the participants mentioned the pandemic as a barrier to adopting a healthy lifestyle. While some participants spoke about the importance of their own 'willpower' in maintaining their exercise regimen during the pandemic, they acknowledged the challenge posed by the pandemic. Other studies have similarly reported dramatic lifestyle changes in reducing physical activity with increased sedentary behaviours and reduced physical activity during the Covid-19 pandemic [39, 40]. These unhealthy lifestyle behaviours observed in the pandemic can potentially lead to the persistence of these poor lifestyle habits and the development of chronic diseases. Contact tracing mobile Apps were rolled out very early during the COVID-19 pandemic, in Singapore, mainly as a means of infection control [41]. However, at the national level, there was no imperative to develop or

implement apps that could provide information on healthy lifestyles during the period of enforced social isolation. A conversational agent such as Elena+ [42], which provided coaching sessions, behavior change activities, and intention/goal formation to promote a healthy lifestyle during the pandemic, must be culturally adapted and implemented globally. Such digital health interventions are of value both during the pandemic and beyond it to ensure that at-risk populations are engaged in health promotion [42].

This study identified several cultural factors as barriers to adopting a healthy lifestyle. Prevalence of diabetes varies among Chinese, Malay, and Indian ethnicities [16], and it is often ascribed to dietary differences, especially in the popular media. Interestingly, while some participants of Indian and Malay ethnicities said that their food choices and food preparation might be high in calories, many Chinese participants also commented that traditional Malay or Indian food was too sweet or oily. However, most of our participants acknowledged cross-cultural eating and said they preferred deep-fried or sweet food. They also felt that they ended up eating more of it than low-fat options that were often not spicy or tasty. All the major festivals celebrated in Singapore were identified as periods where people choose not to count calories and enjoy feasting with friends and families, highlighting the importance of traditional food during social gatherings and religious or traditional celebrations. A study on South Asian immigrants in Australia identified a similar theme where participants felt that food was a central theme of social gatherings and indicated their preference for traditional food in these settings [43]. Multilevel interventions targeted towards families, i.e., those that involve children and parents, comprising programs that increase knowledge, willingness to try nutritious food, and encourage menu modifications without compromising on taste, could be trialed in Singapore [44]. Given the importance of family in Asia and the role of interpersonal factors as both barriers and facilitators in this population, such an approach may be both appealing and strategic.

Workplace and technology emerged as significant environmental facilitators. The Health Promotion Board, Singapore, has spearheaded workplace initiatives. They identified workplaces as a key setting as most adult Singapore residents spend most of their day at work. The focus areas include obesity prevention and management and chronic disease management. They work proactively with companies to support them with the necessary tools to ensure a health-promoting workplace. These initiatives have resulted in many companies providing workplace talks on physical and mental health, organising group physical activities, providing healthy food alternatives in canteens, subsidising the cost of fruits, and distributing fruits and healthy snacks to staff [45]. Technology has been classified as a component of the physical environment's artificial elements [46]. Given the focus on developing Singapore as a 'smart nation' to leverage technology and implement it nationally, the widespread interest and adoption of technology for a healthy lifestyle were not surprising. With the rapid technological advances and integration of smartphones with wearable devices that can assess physical activity, sedentary behavior, heart rate, and intensity levels of physical activity [47], many people prefer wearables as facilitators of a healthy lifestyle.

Furthermore, technological advancement has resulted in better identification and tracking of previously non-identifiable physical activity (e.g., stair climbing, outdoor cycling), which our participants mentioned as particularly appealing as they catered to their lifestyle. Participants also alluded to the persuasive technology that was often incorporated within the wearables. The ability to send their achievements (hours exercised, distance covered, etc.) to online communities or friends with whom they could compete, stay accountable or get encouragement for their achievements was seen as a facilitator by some participants. At the same time, others mentioned the reminders to pause and take deep breaths as being useful in the middle of stress-filled days. Persuasive technology is defined as technology that is designed to change individuals' attitudes or behaviours through persuasion and social influence, but not through

coercion [48]. Technological advances can be used to nudge individuals to engage in more physical activity. This is done by capturing the data and comparing it with historical data; tracking improvement over time; linking data to social media; and sending encouraging messages to the wearer, such as asking them to move more or taking some time for deep breathing or mindfulness. While these are exciting and transformative developments that facilitate the adoption of a healthy lifestyle, little data is available concerning how successful these apps or wearables are at enabling users to lose weight or get fit over time. Research into the effectiveness of many of these technologies is still in its infancy.

Policy-driven changes to the built environment and education curriculum were identified by participants as facilitators of the adoption of a healthy lifestyle. Our study highlights the importance of positioning health promotion in city planning and developing 'healthy built environments.' Surprisingly, participants did not mention the 'utilitarian walking' encouraged in Singapore due to greater land-use mixes that ensure easy accessibility to various locations in the neighbourhood, like shops, food courts, and primary care services. Instead, they focused mainly on recreational walking enabled by small open places like playgrounds and large parks within walking distance of the residential neighbourhoods. Many pointed out that with other walkability features such as safe sidewalks, covered pathways, and easy accessibility, there was no excuse not to exercise in Singapore. Several other studies in Singapore have similarly established that physical activity levels are closely associated with the built environment characteristics [49, 50].

The incorporation of physical education classes in the school curriculum meets several important objectives. First, it ensures that students participate in appropriate amounts of physical activity during lessons. Secondly, they become equipped with the knowledge and skills to be physically active throughout their life [51]. Several participants referred to the need to incorporate physical activities in the routine right from childhood as they felt that children

who learn these skills would use them lifelong. And that it is more difficult to convince an older adult to do physical activities, especially if they have not done them before. However, schools should consider providing a diverse range of physical activity experiences so that the needs and interests of all children are met. Schools should also consider incorporating healthy eating practices as part of their curriculum to reduce the risk of childhood obesity further and promote lifelong healthy nutritional practices [52].

There are some limitations to our study. Since the study was planned before the COVID-19 pandemic, in the early part of the study, the team did not have specific questions or probes that examined factors that could be related to the pandemic. The pandemic may have also limited those who were not technologically savvy or worried about the impact of the infection from participating in the study. The pandemic experience may have coloured the participants' attitudes towards healthy lifestyles, i.e., they may have had a more positive attitude toward it, given the higher risk of poor Covid-19 infections amongst those with multimorbidity and other lifestyle-associated risk factors. While the study allowed the participants to define what a healthy lifestyle meant to them, the discussion on barriers and facilitators centred mainly around physical activity and nutrition. There was limited discussion around other aspects of a healthy lifestyle, like using tobacco, alcohol, sleep, and mental health. The authors could not link the quantitative and qualitative data due to ethical considerations. During the qualitative phase, we did not collect such data (BMI, smoking habits, etc.), limiting a deeper understanding of the participant's narratives. The strengths of our study include a good representation of people across ethnic groups and languages in a multi-ethnic population. The use of one-to-one interviews that led to frank discussions on barriers and facilitators and the inclusion of data from 30 interviews ensured thematic saturation. The qualitative researchers involved in this study came from different disciplines, thus providing a transdisciplinary understanding of the

phenomenon under study. Lastly, the study results triangulate well with other studies examining barriers and facilitators of healthy lifestyles.

Conclusions

Our study found that participants were aware of the several steps undertaken by the Singapore Government to promote a healthy lifestyle. The school-based and workplace health-promoting activities were seen as promoting and ensuring the adoption of a healthy lifestyle. Participants were also cognisant of the built environment in Singapore that encourages adopting a healthy lifestyle. However, despite these consistent efforts by the Singapore Government, participants identified several barriers to adopting a healthy lifestyle. Personal and interpersonal factors like willpower, self-regulation, and influence from family and peers were identified as important barriers. On the other hand, devices for monitoring activities and diet emerged as significant facilitators that can be further leveraged to improve the health of populations.

Most of the barriers identified are amenable to interventions. Incorporating educational material with motivational techniques, short interventions to improve self-regulation delivered by health care professions, multilevel interventions targeted at families, and nudge technology are ways to promote a healthy lifestyle should be explored in future studies.

Supporting Information

S1 File: Semi-structured interview guide

References

1. Crimmins EM. Lifespan and Healthspan: Past, Present, and Promise. Gerontologist. 2015;55:901–11. doi: 10.1093/geront/gnv130.
2. Centers for Disease Control and Prevention (CDC). Ten great public health achievements-United States, 2001-2010. MMWR Morb Mortal Wkly Rep. 2011;60(19): 619–23.
3. McKeown RE. The epidemiologic transition: Changing patterns of mortality and population dynamics. Am J Lifestyle Med. 2009;3(1 Suppl):19S–26S. doi: 10.1177/1559827609335350.
4. Elwyn G, Laitner S, Coulter A, Walker E, Watson P, Thomson R. Implementing shared decision making in the NHS. BMJ. 2010;341:c5146. doi: https://doi.org/10.1136/bmj.c5146
5. Resnik DB. Responsibility for health: personal, social, and environmental. J Med Ethics. 2007 Aug;33(8):444-5. doi: 10.1136/jme.2006.017574.
6. World Health Organisation. What is a healthy lifestyle? EUR/ICP/LVNG 01 07 02. E66134. WHO Regional Office for Europe, Copenhagen; 1999.
7. Pender NJ, Murdaugh CL, Parsons MA. Health Promotion in Nursing Practice. 5th ed. Upper Saddle River, NJ: Prentice-Hall; 2006.
8. Cardel MI, Szurek SM, Dillard JR, Dilip A, Miller DR, Theis R, et al. Perceived barriers/facilitators to a healthy lifestyle among diverse adolescents with overweight/obesity: A qualitative study. Obes Sci Pract. 2020;6:638–48. doi: 10.1002/osp4.448.
9. Murray J, Craigs CL, Hill KM, Honey S, House A. A systematic review of patient-reported factors associated with uptake and completion of cardiovascular lifestyle

behaviour change. BMC Cardiovasc Disord. 2012;12:120. https://doi.org/10.1186/1471-2261-12-120.

10. Kelly S, Martin S, Kuhn I, Cowan A, Brayne C, Lafortune L. Barriers and facilitators to the uptake and maintenance of healthy behaviours by people at mid-life: A rapid systematic review. PLoS ONE. 2016;1(1):e0145074. doi:10.1371/journal.pone.0145074.

11. Perera M, Arambepola C, Gillison F, Peacock O, Thompson D. Perceived barriers and facilitators of physical activity in adults living in activity-friendly urban environments: A qualitative study in Sri Lanka. PLoS One. 2022;17(6):e0268817. doi: 10.1371/journal.pone.0268817.

12. Shrestha A, Pyakurel P, Shrestha A, Gautam R, Manandhar N, Rhodes E, et al. Facilitators and barriers to healthy eating in a worksite cafeteria: a qualitative study from Nepal. Heart Asia. 2017;9(2):e010956. doi: 10.1136/heartasia-2017-010956.

13. Chandra A, Nongkynrih B. Facilitators and barriers of physical activity in prevention and control of NCD-A qualitative study in North India. J Trop Med Health. 2019;3:144. doi: 10.29011/JTMH-144. 000044

14. Department of Statistics, Singapore. Census of population, population trends, 2021. Available: https://www.singstat.gov.sg/-/media/files/publications/population/population2018.pdf [Accessed 5th March 2022]

15. World Bank. Data: GDP per capita (current US$) [Last assessed 5 March 2022]. Available from http://data.worldbank.org/indicator/NY.GDP.PCAP.CD.

16. Ministry of Health and Health Promotion Board, Singapore Epidemiology & Disease Control Division and Policy, Research & Surveillance Group. National Population Health Survey 2020. Available online at https://www.moh.gov.sg/docs/librariesprovider5/default-document-library/nphs-2020-survey-report.pdf Last accessed on 4th May 2022.

17. Sloan RA. Moving towards an active living society. A view from Singapore. ASPETAR Sports Med J. 2015;4:248–53.

18. Trost SG, Blair SN, Khan MK. Physical inactivity remains the greatest public health problem of the 21st century: evidence, improved methods and solutions using the ‘7 investments that work’ as a framework. Brit J Sport Med. 2014;48:169–70. doi: 10.1136/bjsports-2013-093372.

19. Nanditha A, Ma RC, Ramachandran A, Snehalatha C, Chan JC, Chia KS, et al. Diabetes in Asia and the Pacific: Implications for the Global Epidemic. Diabetes Care. 2016;39: 472–85. doi: 10.2337/dc15-1536.

20. Ministry of Health, Singapore. Update on the war on diabetes. MOH COS 2020 Factsheet. Available online at https://www.moh.gov.sg/docs/librariesprovider5/cos2020/cos-2020---update-on-war-on-diabetes.pdf. Last accessed on 11th August 2022.

21. Hill JO, Galloway JM, Goley A, Marrero DG, Minners R, Montgomery B, et al. Scientific statement: Socio-ecological determinants of prediabetes and type 2 diabetes. Diabetes Care. 2013;36:2430–9. doi: 10.2337/dc13-1161.

22. McLeroy KR, Bibeau D, Steckler A, Glanz K. An ecological perspective on health promotion programs. Health Educ Q. 1988;15:351–37. doi: 10.1177/109019818801500401.

23. AshaRani PV, Abdin E, Kumarasan R, Siva Kumar FD, Shafie S, Jeyagurunathan A, et al. Study protocol for a nationwide Knowledge, Attitudes and Practices (KAP) survey on diabetes in Singapore’s general population. BMJ Open. 2020;10(6):e037125. doi: 10.1136/bmjopen-2020-037125.

24. Sandelowski M. Focus on quantitative methods: Sample sizes, in qualitative research. Res Nurs Health. 1995;18:179–83.

25. Jepson R, Harris FM, Bowes A, Robertson R, Avan G, Sheikh A. Physical activity in South Asians: an in-depth qualitative study to explore motivations and facilitators. PLoS One. 2012;7(10):e45333. doi: 10.1371/journal.pone.0045333.

26. Donaldson-Feilder E, Lewis R, Pavey L, Jones B, Green M, Webster A. Perceived barriers and facilitators of exercise and healthy dietary choices: A study of employees and managers within a large transport organisation. Health Educ J. 2017;76(6):661–75.

27. Kvale S, Brinkmann S. Interviews. In: Learning the Craft of Qualitative Research Interviewing. Sage; 2009.

28. Ritchie J, Spencer L. Qualitative data analysis for applied policy research. In A. Bryman & R. Burgess (Eds.), Analyzing qualitative data (pp. 305–329). Routledge; 1994.

29. Ward DJ, Furber C, Tierney S, Swallow V. Using Framework Analysis in nursing research: a worked example. J Adv Nurs. 2013;69(11):2423–31. doi: 10.1111/jan.12127.

30. Srivastava A, Thomson SB. Framework Analysis: A Qualitative Methodology for Applied Policy Research. J Admin Gov. 2009;4(2):72–9.

31. Spencer, L. Ritchie, J., O'Connor, W., Morrell, G., & Ormston, R. Analysis in practice. In J. Ritchie, J. Lewis, C. McNaughton Nicholls, & R. Ormston (Eds.), Qualitative research practice, 2nd ed., pp. 282. London, UK: SAGE; 2014

32. McMorrow L, Ludbrook A, Macdiarmid JI, Olajide D. Perceived barriers towards healthy eating and their association with fruit and vegetable consumption. J Public Health. 2017;39:330–8. doi: 10.1093/pubmed/fdw038.

33. Wongprawmas R, Sogari G, Menozzi D, Mora C. Strategies to Promote Healthy Eating Among University Students: A Qualitative Study Using the Nominal Group Technique. Front Nutr. 2022;9:821016. doi: 10.3389/fnut.2022.821016.

34. Justine M, Azizan A, Hassan V, Salleh Z, Manaf H. Barriers to participation in physical activity and exercise among middle-aged and elderly individuals. Singapore Med J. 2013; 54(10):581–6. doi: 10.11622/smedj.2013203.

35. Tsukayama E, Toomey SL, Faith MS, Duckworth AL. Self-control as a protective factor against overweight status in the transition from childhood to adolescence. Arch Pediatr Adolesc Med. 2010;164(7):631–5. doi: 10.1001/archpediatrics.2010.97.

36. Smith T, Panfil K, Bailey C, Kirkpatrick K. Cognitive and behavioral training interventions to promote self-control. J Exp Psychol Anim Learn Cogn. 2019;45(3):259–79. doi: 10.1037/xan0000208.

37. Stadler G, Oettingen G, Gollwitzer PM. Intervention effects of information and self-regulation on eating fruits and vegetables over two years. Health Psychol. 2010;29(3): 274–83. doi: 10.1037/a0018644.

38. Stadler G, Oettingen G, Gollwitzer PM. Physical activity in women: effects of a self-regulation intervention. Am J Prev Med. 2009;36(1):29–34. doi: 10.1016/j.amepre.2008.09.021.

39. Giuntella O, Hyde K, Saccardo S, Sadoff S. Lifestyle and mental health disruptions during COVID-19. Proc Natl Acad Sci USA. 2021;118(9):e2016632118. doi: 10.1073/pnas.2016632118.

40. Caroppo E, Mazza M, Sannella A, Marano G, Avallone C, Claro AE, et al. Will nothing be the same again?: Changes in lifestyle during COVID-19 pandemic and consequences on mental health. Int J Environ Res Public Health. 2021;18(16):8433. doi: 10.3390/ijerph18168433.

41. Lee T, Lee H. Tracing surveillance and auto-regulation in Singapore:‘smart’responses to COVID-19. Media Int. Aust. 2020;177:47–60

42. Ollier J, Neff S, Dworschak C, Sejdiji A, Santhanam P, Keller R, et al. Elena+ Care for COVID-19, a Pandemic Lifestyle Care Intervention: Intervention Design and Study Protocol. Front Public Health. 2021;9:625640. doi: 10.3389/fpubh.2021.625640.
43. Nisar M, Khan A, Kolbe-Alexander TL. 'Cost, culture and circumstances': Barriers and enablers of health behaviours in South Asian immigrants of Australia. Health Soc Care Community. 2022; 18th February. doi: 10.1111/hsc.13759.
44. Blue Bird Jernigan V, Taniguchi T, Haslam A, Williams MB, Maudrie TL, Nikolaus CJ, et al. Design and methods of a participatory healthy eating intervention for indigenous children: The FRESH Study. Front Public Health. 2022;10:790008. doi: 10.3389/fpubh.2022.790008.
45. Health Promotion Board. Workplace 2020. Available online at https://www.hpb.gov.sg/workplace
46. Gadais T, Boulanger M, Trudeau F, Rivard MC. Environments favorable to healthy lifestyles: A systematic review of initiatives in Canada. J Sport Health Sci. 2018;(1):7–18. doi: 10.1016/j.jshs.2017.09.005.
47. Graham DJ, Hipp JA. Emerging technologies to promote and evaluate physical activity: Cutting-edge research and future directions. Front Public Health. 2014;2:66. doi: 10.3389/fpubh.2014.00066.
48. Dominic D, Hounkponou F, Doh R, Ansong E, Brighter A. Promoting physical activity through persuasive technology. Int J Invent Eng Sci. 2013;2:16–22.
49. Tao Y, Zhang W, Gou Z, Jiang B, Qi Y. Planning walkable neighborhoods for "Aging in Place": Lessons from five aging-friendly districts in Singapore. Sustainability. 2021; 13(4):1742.

50. Song S, Yap W, Hou Y, Yuen B. Neighbourhood built environment, physical activity, and physical health among older adults in Singapore: A simultaneous equations approach. J Transp Health. 2020;18:100881.

51. Simons-Morton BG. Implementing health-related physical education. In Pate RR and Hohn RC. (eds), Health and Fitness Through Physical Education. Human Kinetics, Champaign, IL, 1994;137–46.

52. Swindle T, Rutledge JM, Selig JP, Painter J, Zhang D, Martin J, et al. Obesity prevention practices in early care and education settings: an adaptive implementation trial. Implementation Sci. 2022;17:25. doi: 10.1186/s13012-021-01185-1.

Figure

Click here to access/download;Figure;Figure 1.jpg

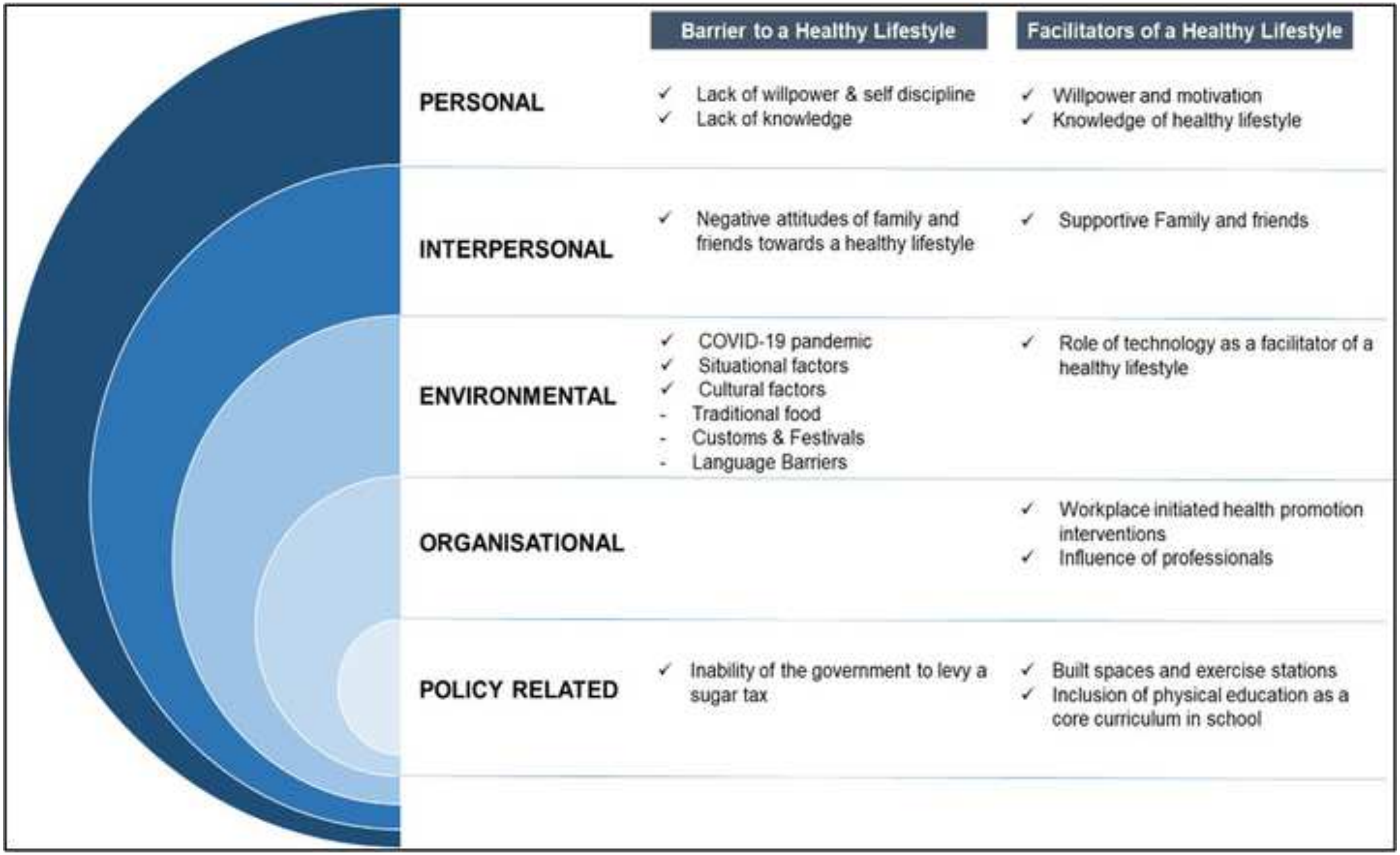

Click here to access/download
Supporting Information
S1 Interview guide.docx

Title: Barriers and Facilitators for adopting a healthy lifestyle in a multi-ethnic population: A qualitative study

Authors: Mythily Subramaniam[1,2], Fiona Devi[1], P.V. AshaRani [1], Yunjue Zhang[1], Peizhi[1], Anitha Jeyagurunathan[1], Kumarasan Roystonn[1], Janhavi Ajit Vaingankar[1], Siow Ann Chong[1]

[1]Research Division, Institute of Mental Health, Singapore

[2]Saw Swee Hock School of Public Health, National University of Singapore

Corresponding Author: Mythily Subramaniam

Assistant Chairman Medical Board (Research)

Research Division, Institute of Mental Health, Buangkok Green Medical Park, 10 Buangkok View, Singapore 539747,

Tel: 65-63893633 Fax: 65-6343 7962, Mythily@imh.com.sg

Abstract

A healthy lifestyle is~~f~~ defined as ’a way of living that lowers the risk of being seriously ill or dying early.’ Although barriers and facilitators of healthy lifestyles have been well-studied among populations like those with chronic non-communicable diseases, adolescents, and older adults in Asia, less information is available on barriers and facilitators perceived by the general adult population. ~~However, despite the awareness that not adopting a healthy lifestyle can result in long term harm, a significant proportion of the population do not adopt a healthy lifestyle which increases the risk of developing chronic illnesses in later life.~~ The current study leveraged the socio-ecological model to gain a deeper understanding of the barriers and facilitators of healthy lifestyle in a sample of Singapore residents using a qualitative methodology. In all [illegible]emi-structured interviews were conducted in English and other local languages from August 2020 to March 2021. Transcripts were analysed using framework analysis. Five main themes pertaining to personal, interpersonal, environmental, socio-cultural, and policy-level factors were classified under the two overarching categories of barriers and facilitators of healthy lifestyles. The results of this study offer important insights into understanding the barriers and facilitators to the adoption of a healthy lifestyle among people in Singapore. Our findings illustrate the complex interplay between individuals, social relationship, environment, and policy that can act as either a barrier or a facilitator to adopting a healthy lifestyle.

Key words: healthy lifestyle; willpower; family; technology; qualitative

Introduction

Life expectancy at birth has increased globally, and improvements in population health status have been observed for every life stage [1]. Public health initiatives such as universal immunisation, enhanced health infrastructure, improved maternal and infant health, tobacco control, and motor vehicle safety have driven these changes globally [2]. This increased life expectancy is associated with changing disease patterns, i.e., a transition from acute to chronic diseases as the primary source of morbidity and mortality worldwide [3]. At the same time, the physician-patient relationship has progressively moved towards shared decision-making, where clinicians and patients make decisions together using the best available evidence [4]. Furthermore, with patients becoming avid consumers of medical knowledge and taking on a more active role in their wellbeing, the responsibility for health rests on individuals and societies [5]. ~~Therefore, while individuals take care of their own health, societies should play an active role in health promotion to potentially prevent chronic diseases like diabetes, coronary artery diseases, and cancers are by adopting a healthy lifestyle such as regular exercise, a good diet, and avoiding smoking and substance use [4,5]~~.

The World Health Organisation (WHO) defines a healthy lifestyle as 'a way of living that lowers the risk of being seriously ill or dying early' [6]. ~~However, despite the awareness that not adopting a healthy lifestyle can result in long-term harm, a significant proportion of the population do things they perceive to be easy or "feel good" in the here and now, which leads to the development of chronic illnesses in later life.~~ Pender's health promotion model defines barriers to a healthy lifestyle as 'factors that directly interfere with the enactment of a health-promoting behavior or mediate by reducing the commitment to the plan of action for changing behavior' [7]. On the other hand, facilitators are defined as factors that promote or enable the uptake and maintenance of a healthy lifestyle [8].

A systematic review of 32 studies from 1980 to February 2010 by Murray et al. [9] found that a better understanding of illness, and perception of significant consequences of illness, were strong facilitators that promoted the uptake of lifestyle behavior change interventions. While stress, depression, lack of social support, and transport and commute time problems were significant barriers. Similar findings were reported by Kelly et al. [10] in their rapid systematic review, which examined barriers and facilitators to the uptake and maintenance of healthy behaviors by people in mid-life. Across 28 qualitative studies, 11 longitudinal cohort studies, and 46 systematic reviews, the authors identified several barriers. These included lack of time, access (to transport, facilities, and resources), financial costs, entrenched attitudes and behaviours, low socioeconomic status, and lack of knowledge. In contrast, facilitators included enjoyment and a sense of wellbeing associated with physical activity, health benefits including healthy ageing, social support, clear messages, accessible websites, and previous experience of ill-health.

Although barriers and facilitators of healthy lifestyles have been well-studied among populations like those with chronic non-communicable diseases, adolescents, and older adults in Asia, less information is available on barriers and facilitators perceived by the general adult population. A qualitative study from Sri Lanka identified several barriers to adopting physical activity in an urban activity-friendly area. The barriers included competing priorities like work, physical concerns like safety, health concerns such as discomfort, resources including facilities and social support, and lack of understanding of the importance of physical activity for health [11]. A study from Nepal that examined the barriers and facilitators to healthy eating in a worksite cafeteria identified unavailability or high cost of healthy foods, cultural preferences for fried food, and difficulty in changing eating habits as the main barriers. This study identified the availability and affordability of healthy food as the main facilitators of healthy eating [12]. A qualitative study conducted in India among the attendees of an urban health centre identified

motivation/willpower, time management skills, knowledge and perceived benefits of physical activity, health problems, and availability of exercise facilities as facilitators of physical activity. T[illegible]ck of time, space, or equipment, unfavourable weather, physical restriction, and laziness were important barriers [13]

~~Several theoretical frameworks have been offered to explain health behavior. The socio-ecological model has been widely used to understand the interrelations between personal, social, and environmental determinants of lifestyle behavior [10-12]. The model is attractive as it incorporates a wide range of influences at multiple levels, and variables included in ecological models comprise intrapersonal or personal (biological, psychological), interpersonal/cultural, organizational, physical environment (built, natural), and policy (laws, regulations,).~~

Singapore is a densely populated, urbanised, city-state in Southeast Asia with a multiracial [illegible]pulation of about 5.5 million, comprising Chinese, Malays, Indians, and a smaller proportion of other ethnicities [14]. The country has a highly developed free-market economy and gross domestic product per capita of about US$60,000 in 2020 [15]. Between 1990 and 2017, life expectancy in Singapore rose from about 76.1 years to 84.8 years; however, healthy life expectancy at birth increased only by 7.2 years, to 74.2 years (16). ~~Singapore has a higher prevalence of diabetes than the global prevalence rate, with nearly one in ten Singaporeans (9.5%) suffering from the disease [15]. At the same time, the total economic cost for working-age patients with diabetes in Singapore, which was USD 787 million in 2010, has been projected to rise to USD 1867 million in 2050 [16], making diabetes a significant public health concern.~~

The Singapore Government has focused on promoting and providing health beyond healthcare facilities. It has made concerted efforts and worked consistently across sectors to promote evidence-based practices to build and maintain a culture of active living in the population [17].

Drawing upon the 7 Investments that Work (7IW) framework [18], the government has worked on transport policies, public education, healthcare, and urban design initiatives to promote physical activity. In addition, Singapore has encouraged the philosophy of individual responsibility, i.e., personal responsibility remains a central tenet of Singapore's approach to healthcare (16).

Furthermore, in 2016, the Singapore Health Minister declared War on Diabetes (WoD). The effort was mainly in response to the higher prevalence of diabetes in Singapore compared to the global prevalence rate, with nearly one in ten Singaporeans (9.5%) suffering from the disease [19]. The WoD had three main thrusts, with the first thrust being prevention through healthy living. As part of this thrust, the government increased the availability and accessibility of physical activity programmes, launched nationwide physical activity-based challenges, and increased the availability of healthier food options in schools, restaurants, and food courts [20]. Thus, against this backdrop of sustained health-promoting policies and the focus on personal responsibility for health, Singapore presents a unique opportunity to understand the barriers and facilitators for the uptake of a healthy lifestyle.

Several theoretical frameworks have been offered to explain health behavior. The socio-ecological model has been widely used to understand the interrelations between personal, social, and environmental determinants of lifestyle behavior [21,22]. The model is attractive as it incorporates a wide range of influences at multiple levels, and variables included in ecological models comprise intrapersonal or personal (biological, psychological), interpersonal/cultural, organizational, physical environment (built, natural), and policy (laws, regulations). We adopted the perspective of the SEM framework to gain a deeper understanding of the barriers and facilitators of a healthy lifestyle in Singapore, given that personal, cultural (multi-ethnic population), and environmental (backdrop of nationwide campaign and initiatives) factors would substantially influence the population's lifestyle. A secondary aim of the study was to

explore if the people were aware of and utilized the interventions launched as part of the WoD to improve healthy lifestyles. The findings of such a study can help develop targeted interventions to overcome the barriers and enhance the facilitators to improve the impact of national campaigns in Singapore.

Methods

Study Design and Setting

The data for the current study was part of a more extensive study that examined the knowledge, attitudes, and protective practices towards diabetes among the general public in Singapore. The study comprised a quantitative survey (n=2895) and a qualitative phase (n=30) to explore the barriers and facilitators of a healthy lifestyle in Singapore. The study methodology was published in an earlier article [23]. The sample for the nationwide survey was derived using a disproportionate stratified sampling design. In all, 12 strata: a combination of 3 strata for ethnicities (Chinese, Malay and Indian) and 4 strata for age (18 to 34 years, 35 to 49 years, 50 to 64 years and 65 years and above) were employed for the sampling. The proportion of respondents in each ethnic group (Chinese, Malay, and Indian) was set at approximately 30%, while the proportion of respondents in each age group was set at around 20% to ensure a sufficient sample size for these population subgroups [23].

The participants for the qualitative study were recruited from among those who participated in the quantitative survey and permitted recontact for future research studies. The inclusion criteria comprised Singapore citizens or permanent residents aged 21 years or above, able to speak in either English, Chinese, Malay, or Tamil, and had not been diagnosed by a doctor as having diabetes. Participants were stratified according to their age (≥ 40 years and < 40 years),

gender, and ethnicity (Chinese, Malay, Indians, and other[illegible] then randomly chosen using an online randomisation software and recruited into the qualitative phase. To account for participant refusals, the sample was drawn in excess (i.e., 60 English-speaking participants and 30 native language speakers). This sampling allows multiple perspectives to be presented that illustrate the complexity of the phenomenon under study [24]. The researchers did not link the quantitative data provided by the individuals with the qualitative data.

Data Collection

In Singapore, a nationwide ‘circuit breaker’ comprising restrictions on public gathering and dining in restaurants, home-based schooling and working, was put in place from 7th April 2020 until 1st June 2020. As of 2nd June 2020, Singapore entered the ‘reopening’ phase, and businesses and activities were progressively allowed to operate. The study period for the current study was eight months, from August 2020 to March 2021. While the measures had been relaxed, and a significant proportion of the population was vaccinated, some participants were uncomfortable doing face-to-face interviews. Interviews were therefore conducted in person or via the Zoom video-conferencing platform, depending on the participants’ preference. In all, 30 interviews were conducted; 20 were in English, four in Chinese, and three in Malay and Tamil. Each interview lasted between 60-90 minutes and was audio-recorded. Participants were interviewed in venues that afforded privacy so ~~th~~they could freely express their views. Participants who opted for a Zoom interview were similarly informed that they should ensure a quiet and private interview setting. Each interview was conducted by a trained qualitative interviewer, who was accompanied by a note-taker, using an interview guide.

The interview guide was developed based on existing literature [25, 26] and further modified after discussions with a general practitioner and a diabetologist. The researchers minimally modified the interview guide to include relevant probes after the first two interviews

(Supplementary File 1). A guided discussion format was used, and participants were encouraged to speak freely about their thoughts and experiences [27].

Data analysis was undertaken concurrently, allowing emerging themes to inform ongoing data collection. The researchers met up regularly and discussed emerging findings to ensure the trustworthiness of the data. Data collection ceased when saturation could reasonably be assumed. The team members transcribed and analysed the data after recruiting the first 20 English-language interviews before commencing the local-language interviews. This was to ensure that we had reached thematic saturation with the data collection and to simultaneously assess the language-specific interviews for the emergence of any new themes. An additional ten interviews were conducted with native speakers, i.e., those able to conver[illegible]nly in Chinese, Malay, and Tamil. This ensured that the perspectives of those belonging to a different socio-cultural background were taken into consideration.

Written informed consent was taken from all the participants, and ethical approval for the study was obtained from the relevant institutional review board (National Healthcare Group Domain Specific Review Board; protocol ref:2019/00926).

Qualitative analysis

The interviews (English and language-specific) were transcribed or translated and transcribed verbatim by a professional transcription firm and checked for accuracy by a study team member. Transcripts were analysed using framework analysis. The Framework Method was originally developed for use in large-scale policy research [28], however it is now used widely in healthcare research. It is a data analysis method rather than a research paradigm which unlike entirely inductive and iterative approaches may be shaped by existing ideas and is less focused on producing a new theory [29]. ~~Framework analysis uses an organized structure of both inductively- and deductively-derived themes [20] to conduct the analysis using a combination of data description and abstraction.~~

Data Familiarisation

First, seven researchers (ZYJ, AR, FD, WP, KR, AJ, and MS) familiarised themselves with the first eight transcripts by reading them multiple times. Through this deep engagement with the data and making notes about key themes, the researcher begins to understand major themes in the data. This was in line with Srivastava and Thomson (30) who stated that, given the large volume of data in qualitative research, not every piece of material may be reviewed at this stage. Following the deep reading, initial themes were identified by individual researchers. These themes were checked against the interview guide and study objectives, resulting in the development of a set of preliminary codes for different barriers and facilitators to a healthy lifestyle.

Constructing an initial thematic framework

The framework was built using a combination of a priori (SEM) and emergent themes [21]. The researchers then met to discuss and combine their preliminary codes. These discussions helped in resolving disagreements in defining or including themes. Largely there was consensus among the team members but when there was a disagreement that could not be resolved, the first author made a call on the inclusion and definition of initial themes. These initial codes were then sorted into hierarchy of themes and sub-themes to construct an initial framework for use across the data set. To ensure all research objectives were met, in this case the initial framework consisted of four main categories, with a number of sub-themes for each category.

On reaching a consensus, a codebook was constructed, which contained a detailed description of each code, the inclusion and exclusion criteria, and typical and atypical exemplars to assist with valid and reliable code application.

Indexing and sorting

Each semi structured interview (SSI) was used as a unit of analysis. To determine 'what parts of the data are about the same thing and belong together' (31), labels were applied to 'chunks' of data with the same meaning to decide the category/ subtheme from the framework to assign the text to. Using NVivo, the selected (highlighted) text was 'dragged and dropped' into the relevant sub-themes. This process was followed for all the transcripts.

Three interviewers (AR, FD, and WP) systematically applied the framework to all the transcripts, after achieving an inter-rater reliability of 0.87 (Kappa (κ) value) with the first two transcripts. There were no major disagreements between the three coders on any of the subthemes or category.

Mapping and Interpretation

The finalized themes and subthemes were grouped together. Once the main themes and sub-themes were reviewed and finalised, a matrix was created for each theme, with individual columns for the sub-themes, using Excel.~~We charted the themes with a summary of the main descriptive comments and developed an explanatory account.~~ The first column of the matrix contained case identification details (demographics), followed by summaries of individual themes in subsequent columns. Representative quotes were selected from the SSI to illustrate key themes and subthemes. These themes and subthemes are represented pictorially in Figure 1.

All analyses were conducted using Nvivo V.11 (QSR International. NVivo V.11).

Results

A total of 30 participants (14 females and 16 males) took part in the study. The mean age of participants was 44.7 years (SD=14.7) ranging from 21 to 75 years (Table 1).

Table 1: Socio-demographic profile of participants

Subject ID	Age	Ethnicity	Gender	Language of interview	Education	Employm[illegible]
SS001	25	Chinese	F	English	Diploma	Employed
SS002	24	Chinese	M	English	Diploma	Student
SS003	39	Malay	F	English	Diploma	Employed
SS004	38	Malay	F	English	Post graduate degree	Homemaker/ Housewife
SS005	54	Chinese	F	English	Degree	Employed
SS006	25	Malay	M	English	Diploma	Employed
SS007	34	Malay	M	English	Completed secondary education	Employed
SS008	27	Malay	M	English	Degree	Employed
SS009	31	Malay	F	English	Degree	Employed
SS010	22	Chinese	M	English	Completed high school (equivalent)	Student
SS011	53	Chinese	F	English	Completed high school (equivalent)	Employed
SS012	55	Malay	F	English	Completed primary education	Employed
SS013	56	Chinese	M	English	Diploma	Unemployed
SS014	26	Chinese	F	English	Degree	Employed
SS015	46	Others	F	English	Post graduate degree	Employed
SS016	38	Chinese	M	English	Vocational Institute Training	Unemployed
SS017	24	Others	M	English	University degree	Employed
SS018	58	Indian	M	English	Completed secondary education	Employed
SS019	52	Indian	M	English	Post graduate degree	Employed
SS020	54	Indian	M	English	Degree	Employed
SS021	60	Chinese	M	Chinese	Some secondary education	Employed
SS022	39	Chinese	M	Chinese	Degree	Employed
SS023	60	Chinese	M	Chinese	Completed secondary education	Unemployed
SS024	59	Chinese	F	Chinese	Completed secondary education	Employed
SS025	54	Malay	F	Malay	Completed primary education	Unemployed
SS026	71	Malay	M	Malay	Completed primary education	Homemaker/ Housewife
SS027	65	Malay	F	Malay	Some secondary education	Homemaker/ Housewife
SS028	37	Indian	M	Tamil	Completed secondary education	Employed
SS029	61	Indian	F	Tamil	Completed primary education	Employed
SS030	53	Indian	F	Tamil	Some secondary education	Homemaker/ Housewife

Formatted Table

Five main themes pertaining to personal, interpersonal, environmental, socio-cultural, and policy-level factors were classified under the two overarching themes of barriers and facilitators of healthy lifestyles. The personal, interpersonal, environmental, and policy-level factors comprised subthemes and are highlighted below. Figure 1 shows the summary of the findings of the themes and sub-themes. Minimally edited verbatims have been included that preserve and highlight the participants' experiences and beliefs. The details of the participants have been provided in brackets as Subject ID/Age/ Gender (**M**ale or **F**emale).

Barriers to a Healthy Lifestyle

Personal Factors

Personal factors comprised two main subthemes, which explained the barriers to adopting a healthy lifestyle. These included:

Lack of willpower and self-discipline

About one-third of participants mentioned the lack of willpower as an important barrier to maintaining a healthy lifestyle in terms of diet and exercise. They described people as 'being lazy,' using 'tired,' 'too busy,' and 'work' as an excuse not to partake in physical activity. They also alluded to personal dietary preferences such as liking sweets and desserts and people not having the willpower to resist them. A few of the respondents felt that people were aware of the ill effects of consuming too much sugar, yet they did not have the self-discipline to limit their intake. Respondents similarly felt that in general, people knew about the negative outcomes associated with smoking and alcohol. Yet, they did not quit smoking/ drinking as they did not have the willpower to do it and instead made-up excuses when asked to quit. Respondents also felt that while people may have good intentions and want to adopt a healthy lifestyle, they lacked the willpower to follow through and fell back to unhealthy habits.

"It is your own self-cultivation and self-discipline that has to do with your health. If you don't have good self-discipline and you mess around, what kind of healthy body will you have?" (SS022/39/M)

"It's really hard to change. I have been in contact with a few of them (referring to smokers). One of them stopped smoking for three months. "I stopped smoking." After a few days, he started again. I asked him, 'What do you smoke for?' "Oh, pressure." Pressure is fake." (SS021/60/M)

Lack of knowledge

Participants felt that it was difficult for someone with a chronic health condition to exercise. They also felt that as a person becomes older, they should avoid vigorous exercise as they could injure themselves more easily. Thus, they thought it was advisable for older people and those with chronic health conditions to cut down on their exercise. They did not seem aware that both these groups could exercise safely and substitute high-impact activities for lower-impact ones. Regarding diet, respondents expressed their frustration with contradictory messages on what was healthy. They felt that food that was once considered healthy was no longer believed to be healthy and vice versa. Thus, they were unsure what should be consumed and what should be avoided. A few respondents identified social media as a significant source of unreliable health information.

"For those who have health conditions, it is very difficult for them to do exercise daily. They are in a life situation where they just cannot take part in a lot of things." (SS030/53/F)

"... especially those messages on social media and WhatsApp about your health. Some people say, "Don't take coconut." Some people say, "Yeah. Coconut is healthy. You take more coconut." So really difficult to judge which is right or which is wrong." (SS019/52/M)

Interpersonal Factors

Negative attitudes and negative influences by family and friends towards a healthy lifestyle

Interpersonal factors were mainly identified as the negative attitudes of family and friends towards a healthy lifestyle or the influence of unhealthy practices of friends and family members. E.g., participants mentioned over-eating during family occasions as there was an excess of food and family members or friends urged them to eat more during social gatherings. Participants said that friends who ridiculed their healthy eating habits were barriers to adopting a healthy lifestyle. Several participants shared that when they have food with friends, they tend to over-eat or eat food that is not particularly healthy. They felt uncomfortable not eating the food as they were afraid to be perceived as spoilsports if they did not partake in the feasting and drinking.

"I think there's a stigma against healthy food. I know how some of my friends say that eating your salad is girly or whatnot. So maybe eating salad is associated with teenage girls, I guess. I don't know. And I guess maybe if they smoke a stick or drink beer, it is more associated with masculine ideals. So maybe if you don't follow the party or whatnot, you may be viewed as an outlier or something. So yeah. I would say there is some social stigma tied to certain types of food or lifestyle, I guess." (SS010/22/M)

"…for example, go out with friends and drink milk tea (referring to bubble milk tea, a sweet tea with tapioca balls that is very popular in Singapore) together, and then he drinks a cup and buys you a cup. Will you not drink it? Sometimes, it's not very polite to say no to your friends." (SS022/39/M)

Environmental factors

As most of the interviews were conducted during the COVID-19 pandemic when outdoors activity was restricted, it was not surprising that participants mentioned the pandemic as a significant barrier to physical activity. The fear of infection, as well as safe distancing measures and other restrictions hindered participants from performing outdoor activities and those conducted in gyms or enclosed spaces. The other sub-theme that emerged, pertained to situational factors such as conflicting demands leading to time constraints and the low cost and ready availability of fast food that were perceived to be barriers to adopting a healthy lifestyle.

COVID-19 pandemic

All the participants mentioned the impact of the COVID-19 pandemic on their lifestyles and expressed their fears and frustrations. For example, participants talked about how the social distancing regulations, closure of indoor gyms and training spaces, and the need to mask up (even in outdoor spaces in Singapore) were significant barriers to exercising in Singapore. Moreover, putting a mask on for most of the day in Singapore's hot and humid weather left them feeling tired, irritable, and reluctant to exercise.

"Actually, before COVID, my friends and I played soccer on a weekly basis, every Sunday. Yeah. But because of COVID, then we stopped completely." *(SS008/27/M)*

"And looking at the number of cases (Covid cases), they went up higher. So it's a deterrence to exercising." (SS022/39/M)

Situational factors

The most discussed barrier was time constraints associated with competing priorities such as employment, household chores, and looking after children or older parents. However, other factors such as financial constraints and limited access to healthy food were also reported to impact healthy lifestyle behaviours.

Participants mentioned that healthy food was both expensive and not readily available. They acknowledged that fast food was the most convenient food, and while they knew that consuming a diet rich in calories was associated with being overweight, they were unable to avoid it. The easy availability of fast food at all hours and food stalls that stayed open even during the night in Singapore also encouraged poor food habits. Interestingly, food delivery was associated with an unhealthy lifestyle. Participants felt that food delivery led to people ordering more due to its convenience and easy availability, in addition to that it also led to them eating at odd hours.

While most participants acknowledged that Singapore had several parks and exercise areas that were conducive to physical activity, three of the respondents had concerns about the safety of these facilities. These included dimly lit parks that made it difficult to walk in the night, sharing of the same path by pedestrians, cyclists, and children and teenagers who tended to run or skate, thus increasing the risk of accidents among older adults. In addition, two participants highlighted the lack of good cycling tracks in Singapore, which does not encourage a cycling culture unlike Denmark or the Netherlands.

"Because sometimes, frankly speaking, children are young, and it is hard for you to have any free time for yourself." (SS024/59/F)

"I think maybe the fact that healthy food is quite expensive in Singapore. So I guess food, in general, can be quite affordable if you go hawker center (hawker centres are open-air complexes with many stalls that sell a wide variety of affordably priced food) *or whatnot. But then they are generally not very healthy, so it can be quite troublesome for some people to cook healthier food."* (SS010/22/M)

"Now you can even use your phone to just order, and they will deliver it directly to your house. Ordering food or eating outside food has become so easy that it has become a part of their

lifestyle. So they don't give much consideration to the food itself, and a healthy lifestyle is lost." (SS030/53/F)

Cultural factors

Several cultural factors emerged as barriers to the adoption of healthy lifestyles. Given the multi-ethnic nature of Singapore, barriers pertaining to cultural factors were identified both by people belonging to that ethnocultural group and others. These included:

The cultural importance of traditional food

Participants acknowledged that cooking and eating traditional food was an important ritual in Singapore. However, about one-third of the participants felt that Indian and Malay food tended to be oily and calorie-rich. They also acknowledged that these types of food appealed to people's tastes, and they ended up overeating it. In addition, they felt that desserts unique to these cultures were similarly sweet and not healthy. Coconut milk in traditional food preparations was similarly identified as an unhealthy but a necessary ingredient. Some had also commented, that those belonging to the Chinese ethnicity liked to eat pork and were not willing to switch to healthier meat alternatives (such as white meat). They also acknowledged that Chinese cuisine could be oily as there are many dishes that are deep-fried.

"In fact, Singapore's food is not only western food, but also Malay food, Indian food, and Chinese food. It doesn't contain as much oil as Chinese cuisine in China. Chinese food has a lot of oil. The Chinese food here, oil and salt, will not be so overused, but it will have a lot of fried things. And your Indian food and Malay food, I believe it will have a lot of sugar, especially Malay food." (SS022/39/M)

Customs and Festivals

Participants also talked about food habits of specific ethnic groups, such as eating dinner late in the night and close to bedtime which they perceived as unhealthy. They also acknowledged

that festive periods were not conducive to maintaining a healthy lifestyle as it was all about meeting friends and families and eating. So one tended to over-eat during such periods.

"As far as we Chinese are concerned, if we talk about the Chinese New year, it may be that everyone eats more…" (SS021/60/M)

Language barriers

People from the minority ethnicities expressed their reluctance to participate in community -based group exercises programs as they felt that they would not be able to understand the instructions as these tend to be conducted in a language that they are not conversant in.

"…that's why I just hate to go to some of these community activities. They are all in English, and I won't understand. Most or all are in English only, so I feel a little uncomfortable because of that." (SS030/50/F)

Policy related factors

Policy-related barriers did not emerge very strongly in this group of participants. However, a few participants expressed their frustration with the reluctance of the government to impose a sugar tax. They felt that sugar caused significant harm to a person's health, but it was not something that could be taxed. They mentioned the 'bubble tea fad' in Singapore, which led to the opening of several shops across the country that sold sweet and calorie-rich drinks. The existing policies could not limit such shops; the government they felt could only advocate and educate people about the potential harms of such food.

"I don't think it's realistic. Because, for example, the government tells people not to smoke, then they increase the tax on cigarettes. Don't drink, and they will add a high tax to wine. But it is impossible to add a high tax on sugar because sugar is a necessity in life. Unlike tobacco and wine, it is not a luxury but a part of the diet. Many people in their daily life use sugar. So you can't, the government can't say, add a high tax to milk tea shops. So I think from the

government's point of view that they can't do many things. They can just advocate." (SS022/39/M)

<u>Facilitators of a Healthy Lifestyle</u>

Personal and Interpersonal factors

Most of the facilitators highlighted by the participants in these two themes were the opposite of those mentioned as barriers, although the absence of a barrier was not necessarily a facilitator. For example, personal facilitators highlighted by the majority of participants were 'willpower and motivation.' Participants talked about how willpower was necessary to exercise regularly and eat healthy food. They also felt that if people knew the impact of a healthy lifestyle on long-term outcomes, they would commit to them. One-third of participants also acknowledged that people with health conditions should continue to exercise and maintain a healthy diet as it can prevent secondary complications. They also highlighted the important role of friends and family members in encouraging and supporting a healthy lifestyle, which helped the participants maintain it.

Organisational/ Institutional factors

Workplace initiated health promotion interventions

The workplace emerged as a significant facilitator of a healthy lifestyle. More than half of the participants who were employed mentioned various workplace initiatives that had helped them to become more physically active. This was mainly through workplace wellbeing initiatives such as distribution of fruits, subsidized fruit bazaars at the place of work, educational sessions on diet and its impact on wellbeing, and group exercise classes like Zumba or Yoga. Many workplaces continued these initiatives even during the pandemic by leveraging on Zoom and other platforms.

"But my workplace, I would say they are trying to endorse the whole healthy lifestyle thing.

So we do have things like a monthly fruits giveaway. So every single month we'll get different kinds of fruit and then staff will explain to us what's the benefit of eating food, or fruit rather. We have staff exercise sessions where you can sign up for yoga or gym sessions or go for a walk." (SS009/31/F)

Influence of healthcare and other professionals

Several participants talked about adopting a healthier lifestyle after their healthcare provider (usually a doctor or dietician) advised them about healthy eating or physical activity. They also spoke of informative media programs that encouraged the adoption of a healthy lifestyle.

"Polyclinics (primary care clinics) or I think if I didn't recall wrongly-- I can't really remember. Is it one of the hospitals or polyclinics my parents visited? They actually have a nutritionist who tells you what to eat." (SS016/38M)

"White meat, the doctor's advice is to eat more white meat instead of red meat because red meat is not good for cholesterol." (SS022/39/M)

Environmental factors

Role of Technology as a facilitator of a healthy lifestyle

All the participants highlighted the role of technology as a facilitator of a healthy lifestyle. However, their understanding of technology was varied. Any source of information like Television, Radio, Internet search engines, channels like YouTube, social media sites, and Apps (mobile applications) was described as technology. The role of technology was mainly seen to be threefold. Firstly as a source of information on diet and exercises (information which could be conveyed via articles, programmes/ shows, talks, etc.), secondly as a guide for doing or aiding training, cooking low-calorie food, etc., and lastly for tracking and monitoring their physical activity, heart rate, sleep, as well as food consumption. Some of the participants alluded to Apps that sent them reminders to breathe deeply, meditate, and walk as helpful.

"App for cycling that's called Strava. I think that's just the only healthy fitness app that I have. So they will keep track of your heartbeat, the distance from one point to another, and the speed of cycling." (SS007/34/M)

"It's MyFitnessPal. Well, it tracks my calorie intake for the day. And it's quite specific. It's quite good. But I think they cannot detect some of the local foods. But other than that, they can track basically whatever that goes in your mouth, yeah, whatever you consume. And you can put in your exercises for the day. So, yes, it will help you calculate your goals, like how many KG you want to lose in a month also, which is quite good." (SS008/24/M)

Policy-related factors

The participants highlighted several policy-related facilitators. These included:

Built spaces and exercise stations

More than half of the participants talked about the availability of neighbourhood parks which provided a safe and convenient place to exercise. Participants mentioned that walks were often organised in their neighbourhoods and that volunteers would encourage them to join in these activities. They were also aware that several activities were conducted in these spaces that one could join at no cost. Some also felt that such group activities motivated them, and they enjoyed doing these more than doing exercises by themselves. Participants also mentioned that there are public swimming pools that one could use and government-run gyms where one could access high-quality equipment at a minimal cost. Participants acknowledged that the government was constantly upgrading parks and gyms, and they could now easily access parks and exercise corners.

"Walking, our government is good, gave us many parks and so many connectors (scenic roads connecting parks where pedestrians can walk). You cannot say that there are no facilities." (SS024/59/F)

"Sometimes they do come and call me to join, like what's that called, social service Community volunteers. When we exercise with other people in a group, it gives us a sort of motivation." (SS030/53/F)

Inclusion of physical education as a core curriculum in school

About a quarter of the participants mentioned that including physical education in schools and getting children to exercise regularly as part of the school curriculum encouraged incorporating exercise into their lifestyle. They also shared that even institutes of tertiary education offered an excellent array of exercise classes/ options, which encouraged the students to continue exercising.

"The education system right now really talks about mental health and physical health. Even their physical education is different. So I think exposure to that will be one of the factors that will enable them to live a healthy lifestyle." (SS009/31/F)

"So when I went to XXX Poly [polytechnic name], I started Muay Thai. And even the gym was affiliated with XXX [polytechnic name]. So I've been with this gym since the dawn of time. This is my first gym, and I've been with them all the way [laughter]." (SS006/25/F)

Figure 1 Barriers and Facilitators of adopting a healthy lifestyle

Discussion

This article explored the barriers and facilitators of a healthy lifestyle perceived and experienced by a multi-ethnic sample of adults in Singapore. Using a framework analysis approach that comprised two major components: creating an analytic framework and applying this analytic framework, we leveraged the SEM [22] model, to gain a deeper understanding of the barriers and facilitators of a healthy lifestyle. The discussion focuses on key themes that lend well to intervention or were unique to this study.

At the personal level, lack of willpower emerged as a key barrier, while being motivated and having the resolve to exercise or not eat sweet or calorie-rich food, despite the challenges, was identified as a facilitator. Willpower, defined as the capacity to exert self-control, has emerged in several studies as a barrier to healthy eating [32,33] and physical activity [34]. In Tsukayama et al.'s [35] prospective longitudinal study, the researchers found that more self-controlled children were less likely to become overweight as they entered adolescence. Cognitive and behavioral interventions have been developed to promote self-regulation [36] and overcome this barrier. For example, a study among women aged 30-50 years showed that a brief intervention combining information with a self-regulation technique led to the maintenance of high consumption of fruits and vegetables 24 months after the intervention. In contrast, the information-only intervention group returned to baseline consumption of fruits and vegetables [37]. Similarly, another study tested an intervention that combined information with cognitive-behavioral strategies on women's physical activity with an information-only intervention. Women who were randomised to the self-regulation and information session were substantially more active than women who participated in only the information session [38]. While the WoD campaign has ensured dissemination of information on healthy lifestyle, there is a need to conduct studies that have an additional component that teaches and promotes self-regulation. If effective such interventions could be scaled up at the population level.

Environmental factors that emerged as barriers and facilitators were unique to this study. The study period coincided with the COVID-19 pandemic, and all the participants mentioned the pandemic as a barrier to the adoption of a healthy lifestyle. While some participants spoke about the importance of their own 'willpower' in maintaining their exercise regimen during the pandemic, they acknowledged the challenge posed by the pandemic. Other studies have similarly reported dramatic lifestyle changes in terms of reducing physical activity with increased sedentary behaviors and reduced physical activity during the Covid-19 pandemic [39,

40]. These unhealthy lifestyle behaviors observed in the pandemic period can potentially lead to the persistence of these poor lifestyle habits and the development of chronic diseases. There is a need to develop and implement interventions to maintain a healthy lifestyle under quarantine and beyond it, paying particular attention to high-risk groups such as individuals with pre-existing physical health problems and older adults. Contact tracing mobile Apps were rolled out very early during the COVID-19 pandemic, in Singapore, mainly as a means of infection control [41]. However, at the national level there was no imperative to develop or implement apps that could provide information on healthy lifestyles during the period of enforced social isolation. A conversational agent such as Elena+ [42] that provides coaching sessions, behavior change activities, and intention/goal formation promote a healthy lifestyle during the pandemic needs to be culturally adapted and implemented globally. Such digital health intervention are of value both during the pandemic and beyond it to ensure that at -risk populations are engaged in health promotion [42].

Several cultural factors emerged as barriers to adopting a healthy lifestyle in this study. Prevalence of diabetes varies among Chinese, Malay, and Indian ethnicities [16], and it is often ascribed to dietary differences, especially in the popular media. It was interesting to note that while some participants of Indian and Malay ethnicities said that their food choices and food preparation might be high in calories, many Chinese participants also commented that traditional Malay or Indian food was too sweet or oily. However, most of our participants acknowledged cross-cultural eating and said they preferred deep-fried or sweet food. They also felt that they ended up eating more of it than low-fat options that were often not spicy or tasty. All the major festivals celebrated in Singapore were identified as periods where people choose not to count calories and enjoy feasting with friends and families, highlighting the importance of traditional food during social gatherings and religious or traditional celebrations. A study on South Asian immigrants in Australia identified a similar theme where participants felt that food

was a central theme of social gatherings and indicated their preference for traditional food in these settings [43]. Multi-level, interventions that are targeted towards families, i.e., those that involve children and parents, comprising programs that increase knowledge, willingness to try nutritious food, as well as encourage menu modifications without compromising on the taste could be trialled in Singapore [44]. Given the importance of family in Asian populations and the role of interpersonal factors as both barriers and facilitators in this population, such an approach may be both appealing and strategic.

Workplace and technology emerged as significant environmental facilitators. The Health Promotion Board, Singapore, has spearheaded workplace initiatives. They identified workplaces as a key setting as most adult Singapore residents spend most of their day at work. The focus areas include obesity prevention and management and chronic disease management. They work proactively with companies to support them with the necessary tools to ensure a health-promoting workplace. These initiatives have resulted in many companies providing workplace talks on physical and mental health, organising group physical activities and providing healthy food alternatives in canteens, subsidised fruit sales, and distributing fruits and healthy snacks to staff [45]. Technology has been classified as a component of the physical environment's artificial elements [46]. Given the focus on developing Singapore as a 'smart nation' to leverage technology and implement it nationally, the widespread interest and adoption of technology for a healthy lifestyle was not surprising. With the rapid technological advances and integration of smartphones with wearable devices that can assess physical activity, sedentary behavior, heart rate and intensity levels of physical activity [47], we found that people in general and especially young people preferred wearables as facilitators of a healthy lifestyle.

Furthermore, technological advancement has resulted in better identification and tracking of previously non-identifiable physical activity (e.g., stair climbing, outdoor cycling) which our

participants mentioned as particularly appealing as they catered to their lifestyle. Participants also alluded to the persuasive technology that was often incorporated within the wearables. The ability to send their achievements (hours exercised, distance covered, etc.) to online communities or friends with whom they could compete, stay accountable or get encouragement for their achievements was seen as a facilitator by some participants. At the same time, others mentioned the reminders to pause and take deep breaths as being useful in the middle of stress-filled days. Persuasive technology is defined as technology that is designed to change individuals' attitudes or behaviors through persuasion and social influence, but not through coercion [48]. Technological advances can be used to nudge individuals to engage in more physical activity. This is done by capturing the data and comparing it with historical data; tracking improvement over time; linking data to social media; and sending encouraging messages to the wearer, such as asking them to move more or taking some time for deep breathing or mindfulness. While these are exciting and transformative developments that facilitate the adoption of a healthy lifestyle, little data is available concerning how successful these apps or wearables are at enabling users to lose weight or get fit over time. Research into the effectiveness of many of these technologies is still in its infancy.

Policy-driven changes to the built environment and education curriculum were identified by participants as facilitators of the adoption of a healthy lifestyle. Our study highlights the importance of positioning health promotion in city planning and developing 'healthy built environments.' Surprisingly, participants did not mention the 'utilitarian walking' encouraged in Singapore due to greater land-use mixes that ensure easy accessibility to various locations in the neighbourhood like shops, food courts, and primary care services. Instead, they focused mainly on recreational walking enabled by small open places like playgrounds and large parks within walking distance of the residential neighbourhoods. Many pointed out that with other walkability features such as safe sidewalks, covered pathways, and easy accessibility, there

was no excuse not to exercise in Singapore. Several other studies in Singapore have similarly established that physical activity levels are closely associated with the built environment characteristics [49, 50].

The incorporation of physical education classes in the school curriculum meets several important objectives. First, it ensures that students participate in appropriate amounts of physical activity during lessons. Secondly, they become equipped with the knowledge and skills to be physically active throughout their life [51]. Several participants referred to the need to incorporate physical activities in the routine right from childhood as they felt that children who learn these skills would use them lifelong. And that it is more difficult to convince an older adult to do physical activities, especially if they have not done them before. However, schools should consider providing a diverse range of physical activity experiences so that the needs and interests of all children are met. Schools should also consider incorporating healthy eating practices as part of their curriculum to further reduce the risk of childhood obesity and promote lifelong healthy nutritional practices [52].

There are some limitations to our study. Since the study was planned before the COVID-19 pandemic, in the early part of the study, the team did not have specific questions or probes that examined factors that could be related to the pandemic. The pandemic may have also limited those who were not technologically savvy or worried about the impact of the infection through interviews, from participating in the study. The experience of te pandemic may have coloured the attitudes of the participants towards healthy lifestyles i.e., they may have had a more positive attitude toward it, given the higher risk of poor Covid-19 infections amongst those with multimorbidity and other lifestyle associated risk factors. While the study allowed the participants to define what a healthy lifestyle meant to them, the discussion on barriers and facilitators centered mainly around physical activity and nutrition. There was limited discussion around other aspects of a healthy lifestyle like using tobacco, alcohol, sleep, and

mental health. The authors were unable to link the quantitative and qualitative data due to ethical considerations and they did not collect such data (BMI, smoking habits etc.) during the qualitative phase which limits a deeper understanding of their narratives. The strengths of our study include a good representation of people across ethnic groups and languages in a multi-ethnic population. The use of one-to-one interviews that led to frank discussions on barriers and facilitators and the inclusion of data from 30 interviews ensured thematic saturation. The qualitative researchers involved in this study came from different disciplines, thus providing a transdisciplinary understanding of the phenomenon under study. The study results triangulate well with those of other studies examining barriers and facilitators of healthy lifestyles.

Conclusions

Our study found that participants were aware of the several steps undertaken by the Singapore Government to promote a healthy lifestyle. The school-based and -workplace health promoting activities were seen as promoting and ensuring the adoption of a healthy lifestyle. Participants were also cognisant of the built environment in Singapore that encourages the adoption of a healthy lifestyle. However, despite these consistent efforts by the Singapore Government, participants identified several barriers to the adoption of a healthy lifestyle. Personal and interpersonal factors like willpower and self-regulation and influence from family and peers were identified as important barriers. On the other hand, devices for monitoring activities and diet emerged as significant facilitators that can be further leveraged to improve the health of populations.

Most of the barriers identified are amenable to interventions. Incorporation of educational material with motivational techniques, short interventions to improve self-regulation delivered by health care professions, multilevel interventions targeted at families as well as the use of nudge technology as a way to promote healthy lifestyle should be explored in future studies.

Supporting Information

S1 File: Semi-structured interview guide

~~Acknowledgement: This study is supported by the Singapore Ministry of Health's National Medical Research Council under its Health Services Research Grant (NMRC/HSRG/0085/2018).~~

References

1. Crimmins EM. Lifespan and Healthspan: Past, Present, and Promise. Gerontologist. 2015;55:901–11. doi: 10.1093/geront/gnv130.

~~1.~~2. Centers for Disease Control and Prevention (CDC). Ten great public health achievements-United States, 2001-2010. MMWR Morb Mortal Wkly Rep. 2011;60(19): 619–23.

~~2.~~3. McKeown RE. The e~~E~~pidemiologic t~~T~~ransition: Changing p~~P~~atterns of m~~M~~ortality and p~~P~~opulation d~~D~~ynamics. Am J Lifestyle Med. 2009;3(1 Suppl):19S–26S. doi: 10.1177/1559827609335350.

~~3.~~4. Elwyn G, Laitner S, Coulter A, Walker E, Watson P, Thomson R. Implementing shared decision making in the NHS. BMJ. 2010;341:c5146. doi: https://doi.org/10.1136/bmj.c5146

~~4. Piepoli, MF, Hoes AW, Agewall S, Albus C, Brotons C, Catapano A, et al; ESC Scientific Document Group 2016 European guidelines on cardiovascular disease prevention in clinical practice: the sixth joint task force of the European Society of Cardiology and other societies on cardiovascular disease prevention in clinical practice (constituted by representatives of 10 societies and by invited experts). Developed with the special contribution of the European Association for Cardiovascular Prevention & Rehabilitation (EACPR). Eur Heart J. 2016;37(29):2315–81.~~

~~5. Lindström J, Ilanne-Parikka P, Peltonen M, Aunola S, Eriksson JG, Hemiö K, et al. Sustained reduction in the incidence of type 2 diabetes by lifestyle intervention: follow-up of the Finnish diabetes prevention study. Lancet. 2006;368(9548):1673–9.~~

5. Resnik DB. Responsibility for health: personal, social, and environmental. J Med Ethics. 2007 Aug;33(8):444-5. doi: 10.1136/jme.2006.017574.

6. World Health Organisation. What is a healthy lifestyle? EUR/ICP/LVNG 01 07 02. E66134. WHO Regional Office for Europe, Copenhagen, ; 1999.

7. Pender NJ, Murdaugh CL, Parsons MA. Health Promotion in Nursing Practice. 5th ed. Upper Saddle River, NJ: Prentice-Hall, ; 2006.

7.8. Cardel MI, Szurek SM, Dillard JR, Dilip A, Miller DR, Theis R, et al. Perceived barriers/facilitators to a healthy lifestyle among diverse adolescents with overweight/obesity: A qualitative study. Obes Sci Pract. 2020;6:638–48. doi: 10.1002/osp4.448.

8.9. Murray J, Craigs CL, Hill KM, Honey S, House A. A systematic review of patient-reported factors associated with uptake and completion of cardiovascular lifestyle behaviour change. BMC Cardiovasc Disord. 2012;12:120. https://doi.org/10.1186/1471-2261-12-120.

10. Kelly S, Martin S, Kuhn I, Cowan A, Brayne C, Lafortune L. Barriers and fFacilitators to the uUptake and mMaintenance of hHealthy bBehaviours by pPeople at mMid-lLife: A rRapid sSystematic rReview. PLoS ONE. 2016;1(1):e0145074. doi:10.1371/journal.pone.0145074.

11. Perera M, Arambepola C, Gillison F, Peacock O, Thompson D. Perceived barriers and facilitators of physical activity in adults living in activity-friendly urban environments: A qualitative study in Sri Lanka. PLoS One. 2022;17(6):e0268817. doi: 10.1371/journal.pone.0268817.

12. Shrestha A, Pyakurel P, Shrestha A, Gautam R, Manandhar N, Rhodes E, et al. Facilitators and barriers to healthy eating in a worksite cafeteria: a qualitative study from Nepal. Heart Asia. 2017;9(2):e010956. doi: 10.1136/heartasia-2017-010956.

9.13. Chandra A, Nongkynrih B. Facilitators and barriers of physical activity in prevention and control of NCD-A qualitative study in North India. J Trop Med Health. 2019;3:144. doi: 10.29011/JTMH-144. 000044

10. Hurowitz JC. Toward a social policy for health. N Engl J Med. 1993;329:130–3.

11.1. Hill JO, Galloway JM, Goley A, Marrero DG, Minners R, Montgomery B, et al. Scientific statement: Socio-ecological determinants of prediabetes and type 2 diabetes. Diabetes Care. 2013;36:2430–9.

12.1. McLeroy KR, Bibeau D, Steckler A, Glanz K. An ecological perspective on health promotion programs. Health Educ Q. 1988;15:351–37.

13.14. Department of Statistics, Singapore. Census of population, population trends, 2021. Available: https://www.singstat.gov.sg/-/media/files/publications/population/population2018.pdf [Accessed 5th March 2022]

14.15. World Bank. Data: GDP per capita (current US$) [Last assessed 5 March 2022]. Available from http://data.worldbank.org/indicator/NY.GDP.PCAP.CD.

16. Ministry of Health and Health Promotion Board, Singapore Epidemiology & Disease Control Division and Policy, Research & Surveillance Group. National Population Health Survey 2020. Available online at https://www.moh.gov.sg/docs/librariesprovider5/default-document-library/nphs-2020-survey-report.pdf Last accessed on 4th May 2022.

17. Sloan RA. Moving towards an active living society. A view from Singapore. ASPETAR Sports Med J. 2015;4:248–53.

15.18. Trost SG, Blair SN, Khan MK. Physical inactivity remains the greatest public health problem of the 21st century: evidence, improved methods and solutions using the '7 investments that work' as a framework. Brit J Sport Med. 2014;48:169–70. doi: 10.1136/bjsports-2013-093372.

Formatted: Left

Field Code Changed

19. Nanditha A, Ma RC, Ramachandran A, Snehalatha C, Chan JC, Chia KS, et al. Diabetes in Asia and the Pacific: Implications for the Global Epidemic. Diabetes Care. 2016;39: 472–85. doi: 10.2337/dc15-1536.

20. Ministry of Health, Singapore. Update on the war on diabetes. MOH COS 2020 Factsheet. Available online at https://www.moh.gov.sg/docs/librariesprovider5/cos2020/cos-2020---update-on-war-on-diabetes.pdf. Last accessed on 11th August 2022.

21. Hill JO, Galloway JM, Goley A, Marrero DG, Minners R, Montgomery B, et al. Scientific statement: Socio-ecological determinants of prediabetes and type 2 diabetes. Diabetes Care. 2013;36:2430–9. doi: 10.2337/dc13-1161.

22. McLeroy KR, Bibeau D, Steckler A, Glanz K. An ecological perspective on health promotion programs. Health Educ Q. 1988;15:351–37. doi: 10.1177/109019818801500401.

16.

17.23. AshaRani PV, Abdin E, Kumarasan R, Siva Kumar FD, Shafie S, Jeyagurunathan A, et al. Study protocol for a nationwide Knowledge, Attitudes and Practices (KAP) survey on diabetes in Singapore’s general population. BMJ Open. 2020;10(6):e037125. doi: 10.1136/bmjopen-2020-037125.

24. Sandelowski M. Focus on quantitative methods: Sample sizes, in qualitative research. Res Nurs Health. 1995;18:179–83.

25. Jepson R, Harris FM, Bowes A, Robertson R, Avan G, Sheikh A. Physical activity in South Asians: an in-depth qualitative study to explore motivations and facilitators. PLoS One. 2012;7(10):e45333. doi: 10.1371/journal.pone.0045333.

18.26. Donaldson-Feilder E, Lewis R, Pavey L, Jones B, Green M, Webster A. Perceived barriers and facilitators of exercise and healthy dietary choices: A study of employees and managers within a large transport organisation. Health Educ J. 2017;76(6):661–75.

19.27. Kvale S, Brinkmann S. Interviews. In: Learning the Craft of Qualitative Research Interviewing. Sage, ; 2009.

20. Goldsmith LJ. Using Framework Analysis in Applied Qualitative Research. Qual Rep. 2021;26(6):2061–76.

28. Ritchie J, Spencer L. Qualitative data analysis for applied policy research. In A. Bryman & R. Burgess (Eds.), Analyzing qualitative data (pp. 305–329). Routledge, ; 1994.

29. Ward DJ, Furber C, Tierney S, Swallow V. Using Framework Analysis in nursing research: a worked example. J Adv Nurs. 2013;69(11):2423–31. doi: 10.1111/jan.12127.

30. Srivastava A, Thomson SB. Framework Analysis: A Qualitative Methodology for Applied Policy Research. J Admin Gov. 2009;4(2):72–9.

21.31. Spencer, L. Ritchie, J., O'Connor, W., Morrell, G., & Ormston, R. Analysis in practice. In J. Ritchie, J. Lewis, C. McNaughton Nicholls, & R. Ormston (Eds.), Qualitative research practice, 2nd ed., pp. 282. London, UK: SAGE; 2014

22.32. McMorrow L, Ludbrook A, Macdiarmid JI, Olajide D. Perceived barriers towards healthy eating and their association with fruit and vegetable consumption. J Public Health. 2017;39:330–8. doi: 10.1093/pubmed/fdw038.

23.33. Wongprawmas R, Sogari G, Menozzi D, Mora C. Strategies to Promote Healthy Eating Among University Students: A Qualitative Study Using the Nominal Group Technique. Front Nutr. 2022;9:821016. doi: 10.3389/fnut.2022.821016.

24.34. Justine M, Azizan A, Hassan V, Salleh Z, Manaf H. Barriers to participation in physical activity and exercise among middle-aged and elderly individuals. Singapore Med J. 2013; 54(10):581–6. doi: 10.11622/smedj.2013203.

Formatted: English (United States)

25.35. Tsukayama E, Toomey SL, Faith MS, Duckworth AL. Self-control as a protective factor against overweight status in the transition from childhood to adolescence. Arch Pediatr Adolesc Med. 2010;164(7):631–5. doi: 10.1001/archpediatrics.2010.97.

26.36. Smith T, Panfil K, Bailey C, Kirkpatrick K. Cognitive and behavioral training interventions to promote self-control. J Exp Psychol Anim Learn Cogn. 2019;45(3):259–79. doi: 10.1037/xan0000208.

37. Stadler G, Oettingen G, Gollwitzer PM. Intervention effects of information and self-regulation on eating fruits and vegetables over two years. Health Psychol. 2010;29(3): 274–83. doi: 10.1037/a0018644.

27.38. Stadler G, Oettingen G, Gollwitzer PM. Physical activity in women: effects of a self-regulation intervention. Am J Prev Med. 2009;36(1):29–34. doi: 10.1016/j.amepre.2008.09.021.

28.39. Giuntella O, Hyde K, Saccardo S, Sadoff S. Lifestyle and mental health disruptions during COVID-19. Proc Natl Acad Sci USA. 2021;118(9):e2016632118. doi: 10.1073/pnas.2016632118.

40. Caroppo E, Mazza M, Sannella A, Marano G, Avallone C, Claro AE, et al. Will nothing be the same again?: Changes in lifestyle during COVID-19 pandemic and consequences on mental health. Int J Environ Res Public Health. 2021;18(16):8433. doi: 10.3390/ijerph18168433.

41. Lee T, Lee H. Tracing surveillance and auto-regulation in Singapore:‘smart’responses to COVID-19. Media Int. Aust. 2020;177:47–60

29.42. Ollier J, Neff S, Dworschak C, Sejdiji A, Santhanam P, Keller R, et al. Elena+ Care for COVID-19, a Pandemic Lifestyle Care Intervention: Intervention Design and Study Protocol. Front Public Health. 2021;9:625640. doi: 10.3389/fpubh.2021.625640.

Formatted: Indent: Left: 0", Hanging: 0.39"

43. Nisar M, Khan A, Kolbe-Alexander TL. 'Cost, culture and circumstances': Barriers and enablers of health behaviours in South Asian immigrants of Australia. Health Soc Care Community. 2022; 18th February. doi: 10.1111/hsc.13759.

~~30.~~44. Blue Bird Jernigan V, Taniguchi T, Haslam A, Williams MB, Maudrie TL, Nikolaus CJ, et al. Design and methods of a participatory healthy eating intervention for indigenous children: The FRESH Study. Front Public Health. 2022;10:790008. doi: 10.3389/fpubh.2022.790008.

~~31.~~45. Health Promotion Board. Workplace 2020. Available online at https://www.hpb.gov.sg/workplace

~~32.~~46. Gadais T, Boulanger M, Trudeau F, Rivard MC. Environments favorable to healthy lifestyles: A systematic review of initiatives in Canada. J Sport Health Sci. 2018;(1):7–18. doi: 10.1016/j.jshs.2017.09.005.

~~33.~~47. Graham DJ, Hipp JA. Emerging technologies to promote and evaluate physical activity: Cutting-edge research and future directions. Front Public Health. 2014;2:66. doi: 10.3389/fpubh.2014.00066.

~~34.~~48. Dominic D, Hounkponou F, Doh R, Ansong E, Brighter A. Promoting physical activity through persuasive technology. Int J Invent Eng Sci. 2013;2:16–22.

~~35.~~49. Tao Y, Zhang W, Gou Z, Jiang B, Qi Y. Planning w~~W~~alkable n~~N~~eighborhoods for “Aging in Place”: Lessons from f~~F~~ive a~~A~~ging-f~~F~~riendly d~~D~~istricts in Singapore. Sustainability. 2021; 13(4):1742.

~~36.~~50. Song S, Yap W, Hou Y, Yuen B. Neighbourhood built environment, physical activity, and physical health among older adults in Singapore: A simultaneous equations approach. J Transp Health. 2020;18:100881.

~~37.~~51. Simons-Morton BG. Implementing health-related physical education. In Pate RR and Hohn RC. (eds), Health and Fitness Through Physical Education. Human Kinetics, Champaign, IL, 1994;137–46.

~~38.~~52. Swindle T, Rutledge JM, Selig JP, Painter J, Zhang D, Martin J, et al. Obesity prevention practices in early care and education settings: an adaptive implementation trial. Implementation Sci. 2022;17:25~~.~~. doi: 10.1186/s13012-021-01185-1.

27 August 2022

Eliana Carraça
Academic Editor
PLOS ONE

Ref: PONE-D-22-13181
Barriers and Facilitators for adopting a healthy lifestyle in a multi-ethnic population: A qualitative study

Dear Dr. Carraca

We would like to thank you and the reviewers for your constructive review. We have addressed the points raised by the reviewers in the revised manuscript in as tracked changes. Our replies to their comments are attached and highlighted in bold for easy reference. The major comments on the pdf have been addressed separately as we are not sure which reviewer has commented on them. The minor comments have been addressed directly in the text as tracked changes.

Academic Editor

The topic of this paper is of interest to the Plos One scientific community. Adherence to healthy lifestyle behaviours remains a challenge, thus exploring which factors might facilitate or hinder the adoption of these behaviours is critical to improve intervention's design and efficacy. Still, the current paper does not show the novelty of this study. This should be made totally clear in all sections of the paper, but most importantly in the introduction and discussion sections. Authors are also advised to be more specific and support all affirmations in the literature.
We have added relevant references and clarified the novel aspects of this study.

The methodology should be clearer, especially regarding the process of development of the instrument and how the framework analysis was conducted.
The methodology has been substantially revised as suggested by the reviewers.
The impact of COVID on recruitment and on this study should be discussed, as these results could be different in a normal, non-COVID, situation. Being the first study on the Singapore population, this should be interpreted with caution and unlikely generalisable. Therefore, avoid using terms that imply that your findings are comprehensive and generalisable to all Singapore population within the same age range.
We have avoided the use of terms that suggest that the findings are comprehensive and generalizable.

Grammatical errors should be carefully revised.
Grammatical errors have been revised.

Journal Requirements

When submitting your revision, we need you to address these additional requirements.

1. Please ensure that your manuscript meets PLOS ONE's style requirements, including those for file naming. The PLOS ONE style templates can be found at

2. Thank you for stating the following in the Acknowledgments Section of your manuscript:

"This study is supported by the Singapore Ministry of Health's National Medical Research Council under its Health Services Research Grant (NMRC/HSRG/0085/2018)."

We note that you have provided additional information within the Acknowledgements Section that is not currently declared in your Funding Statement. Please note that funding information should not appear in the Acknowledgments section or other areas of your manuscript. We will only publish funding information present in the Funding Statement section of the online submission form.

Please remove any funding-related text from the manuscript and let us know how you would like to update your Funding Statement. Currently, your Funding Statement reads as follows:

"SAC received the funding

NMRC/HSRG/0085/2018

This study is supported by the Singapore Ministry of Health's National Medical Research Council under its Health Services Research Grant

The funders had no role in study design, data collection and analysis, decision to publish, or preparation of the manuscript."

Please include your amended statements within your cover letter; we will change the online submission form on your behalf.

We would like to retain the statement as it is in the online submission form. We have removed the statement in the revised manuscript.

3. In your Data Availability statement, you have not specified where the minimal data set underlying the results described in your manuscript can be found. PLOS defines a study's minimal data set as the underlying data used to reach the conclusions drawn in the manuscript and any additional data required to replicate the reported study findings in their entirety. All PLOS journals require that the minimal data set be made fully available. For more information about our data policy, please see.

Upon re-submitting your revised manuscript, please upload your study's minimal underlying data set as either Supporting Information files or to a stable, public repository and include the relevant URLs, DOIs, or accession numbers within your revised cover letter. For a list of acceptable repositories, please see. Any potentially identifying patient information must be fully anonymized.

Important: If there are ethical or legal restrictions to sharing your data publicly, please explain these restrictions in detail. Please see our guidelines for more information on what we consider unacceptable restrictions to publicly sharing data: https://imsva91-ctp.trendmicro.com:443/wis/clicktime/v1/query?url=http%3a%2f%2fjournals.plos.org%2fplosone%2fs%2fdata%2davailability%23loc%2dunacceptable%2ddata%2daccess%2drestrictions&umid=C1935020-E531-7305-AE14-9D67384FA132&auth=6e3fe59570831a389716849e93b5d483c90c3fe4-a54b28680ec209732c5562257d5ed9d73bc73984. Note that it is not acceptable for the authors to be the sole named individuals responsible for ensuring data access.

We will update your Data Availability statement to reflect the information you provide in your cover letter.

Revised data availability statement: The data underlying the results presented in the study are available from the first author Dr Mythily Subramaniam (Mythily@imh.com.sg)

Reviewer: 1

1) As the authors note, many studies have been published describing barriers and facilitators to healthy lifestyle practices. While this study is novel in its focus on Singapore, the results do not seem to differ much at all from the large body of work on this topic. It would be useful in the discussion to frame how the findings of this study compare to other similar studies. Are there barriers and facilitators specific to adults in Singapore that could be targeting in a healthy lifestyle intervention?
We would like to thank the reviewer for this suggestion. We have revised our introduction to address how the study is different from others that have been carried out in other countries.

2) Authors make the claim that this study provides a "comprehensive" understanding of barriers and facilitators (pg 5). I am not sure this claim can be made with a fairly small sample (n=30). The paper can still bring forward important findings without being comprehensive.
We have removed the word 'comprehensive'.

3) Pg 8: spell out "SSI" on first use
We apologise for the error and have added the full form as suggested by the reviewer.

4) Pg 8, table 1: Given the important of education level and adoption of healthy lifestyle

habits (as the authors mention in the introduction), it's surprising that education is not included in the demographics. It would also be interesting to see how barriers and facilitators differed among those with varying levels of education.
We have included the educational backgrounds of the participants in the revised table.

5) Similarly to the comment above, it would have been nice to see a segmentation of the data by demographics. For example, are barriers and facilitators different for men and women? Older and younger people? By ethnicity and/or language?
We are unable to do such an analysis as the study design was set up to get a good representation across the groups but did not set out to examine differences across the groups. The sample size does not lend to such a nuanced segmentation and we apologise for that.

6) pg 14: What is meant by "hawker center" in the quote? Provide definition or use a different quote.
We have explained what is meant by a 'hawker center'. We think it is important to retain this quote as it highlights the availability of affordable food which is often not healthy.

7) pg 16: What is meant by "community exercises"? Is this physical activity opportunities in the community?
We have clarified the term by changing it to community-based group exercise programs for clarity.

8) Informed consent was collected from all participants. Was the study protocol reviewed and approved by an institutional review board?
Yes, we have clearly stated just below the statement on consent that - ethical approval for the study was obtained from the relevant institutional review board (National Healthcare Group Domain Specific Review Board; protocol ref:2019/00926).

9) In the introduction, the authors state that this paper will contribute to developing interventions to promote healthy lifestyles among people in Singapore, where diabetes and other chronic conditions are on the rise. I would therefore expect to see explicit recommendations in the discussion or conclusion for interventions tailored to this context. Most of the recommendations mentioned (PA/healthy eating in schools, built environment to promote PA) have been well studied and documented in other contexts and are not novel or innovative. In the revision, I suggest the authors consider what this data adds to the literature and how it can be used to inform intervention development.
We have revised our discussion and conclusion as suggested by the reviewer.

10) Various grammatical errors were noted throughout the manuscript (see comments in attached PDF). Suggest careful re-reading and editing for clarity.
We have revised the manuscript as suggested by the reviewer.

Reviewer #2:

Please see the review comments in the PDF document. I have highlighted the comments.

Main comments in the pdf

Introduction

1. Authors should take a paragraph or two to summarize the studies in Asia populations. This would help to demonstrate why a specifically cultural focus/regional focus is important.
   We have added a paragraph focusing on Asian studies in the revised manuscript.

2. At some point, authors need to explain why the study reported here is focused on general health behaviors vs. Diabetes related health behaviors, as the parent study is a diabetes study. Otherwise, authors are encouraged to situate these findings within health behaviors around Diabetes.
   As we have stated in the revised manuscript the intent of the study was to examine barriers and faciltators to healthy lifestyle in the broader context of general health behaviors which have been promoted at the policy level in Singapore. While the larger study was towards knowledge, attitudes and practices towards diabetes, the questions on lifestyle were general and not towards diabetes related health behaviors (Koh YS, Asharani PV, Devi F, Roystonn K, Wang P, Vaingankar JA, Abdin E, Sum CF, Lee ES, Müller-Riemenschneider F, Chong SA, Subramaniam M. A cross-sectional study on the perceived barriers to physical activity and their associations with domain-specific physical activity and sedentary behaviour. BMC Public Health. 2022 May 26;22(1):1051. doi: 10.1186/s12889-022-13431-2). This was also in consultation with diabetologists who opined that diabetes was often associated with other comorbid conditions and risk factors for several NCIDs were common. Thus, it was decided to understand general health behaviors. We have clarified this in the methodology.

Methodology

3. Please clarify: was this sampling method used for the parent study AND the interview component or both or just the interview component? If a specific sampling method was conducted (were there only 30 people who agreed to be recontacted from the survey? Probably not). So how did authors select FOR RECRUITMENT those who said they'd be willing to be contacted.
   We have clarified the sampling method for the parent study. For the current study the participants were sampled randomly from those who gave consent for recontact. We have clarified the process in the methodology.

4. Are authors claiming that there were no a priori coding structures? If so, why use framework analysis?

We would like to respectfully state that a framework analysis can be used without apriori coding structures. We have further expanded the steps to explain what we did. While our initial coding was guided by our research questions and hence the topic guide, we did not use apriori coding.
Framework analysis in applied research have varied from highly deductive analysis (Pope et al., 2000) to inductively-oriented approaches in healthcare research (e.g., Goldsmith et al., 2017; Swallow et al., 2011).

Results

5. Specify cis, trans, nonbinary gender participants.
   None of the participants identified themselves as cis/ trans or non-binary.

6. Authors should describe more about the participants in this sample: rurality, occupation (or employment), wealth or income, some measure of health (healthy weight? Health behavior such as smoking?). Readers need to understand more about the sample for this interview cohort.
   We have described the sample in Table 1. Singapore is a highly urbanised city-state, there is no rural area in Singapore. We did not link health data to the qualitative interviews, and we have acknowledged it as a limitation of the study. We have added education and employment data.

Overall, the paper does not show the novelty of this study, even though (as argued but not substantiated) that Singapore has yet to be the focus of such a study.
We have revised our introduction to substantiate the novelty of the study.

If authors proceed with a revision, care and clarification is necessary with the manuscript revision to demonstrate study rigor and allow for replication. Further, authors should revisit the discussion and conclusion to 1) enhance the limitations section, and 2) truly demonstrate why this study is novel and important in PLOS One
We have made the changes suggested by the reviewers.

We hope that we have addressed the reviewers' comments adequately and we look forward to a favourable reply.

Regards

Mythily Subramaniam

Assistant Chairman Medical Board (Research)

Research Division

Institute of Mental Health

Buangkok Green Medical Park
10 Buangkok View
Singapore 539747
Tel: 65-6389 3633, Fax: 65-6343 7962
Mythily@imh.com.sg
