# Supplementary material for: Barriers and facilitators for adopting a healthy lifestyle in a multi-ethnic population: A qualitative study
Source: PLoS One. 2022 Nov 2;17(11):e0277106. doi: 10.1371/journal.pone.0277106 (PMC9629631; doi:10.1371/journal.pone.0277106)
Supplement: Attachment — Submitted filename: Response to Reviewers October.doc [file pone.0277106.s006.doc]

15 October 2022

Eliana Carraça
Academic Editor
PLOS ONE

**Ref:** PONE-D-22-13181R1

**Barriers and Facilitators for adopting a healthy lifestyle in a multi-ethnic population: A qualitative study**

Dear Dr. Carraca

We would like to thank you and the reviewers for the second review. We have addressed the points raised by the reviewers in the revised manuscript as tracked changes. Our replies to their comments are attached and highlighted in bold for easy reference in our reply.

**Academic Editor**

The manuscript was improved, but there are still some issues that need to be properly addressed. Please see the comments in the attached file. Make sure you reply to all comments, including those left in the previous response letter to reviewers.

**We have addressed all the comments in the attached file.**

**Reviewer: 1**

(Comments in pdf file and response to our Reply to the reviewers)

**We want to thank the reviewer for once again going through our article carefully and providing us with invaluable insights. The changes suggested in the language have been incorporated directly as tracked changes in the manuscript.**

**We analysed the themes by socio-demographic groups and presented the data.**

We hope we have addressed the reviewers’ comments adequately and look forward to a favourable reply.

Regards

Mythily Subramaniam

Assistant Chairman Medical Board (Research)

Research Division

Institute of Mental Health

Buangkok Green Medical Park

10 Buangkok View

Singapore 539747

Tel: 65-6389 3633, Fax: 65-6343 7962

Mythily@imh.com.sg
